# In Silico Analyses of a Promising Drug Candidate for the Treatment of Amyotrophic Lateral Sclerosis Targeting Superoxide Dismutase I Protein

**DOI:** 10.3390/pharmaceutics15041095

**Published:** 2023-03-29

**Authors:** Gabriel Rodrigues Coutinho Pereira, Bárbara de Azevedo Abrahim-Vieira, Joelma Freire de Mesquita

**Affiliations:** 1 Bioinformatics and Computational Biology Laboratory, Federal University of the State of Rio de Janeiro—UNIRIO, Rio de Janeiro 22290-250, Brazil; 2 Molecular Modeling and QSAR Laboratory, Federal University of Rio de Janeiro—UFRJ, Rio de Janeiro 21941-590, Brazil

**Keywords:** amyotrophic lateral sclerosis, in silico, molecular dynamics, ADMET prediction

## Abstract

Amyotrophic lateral sclerosis (ALS) is the most prevalent motor neuron disorder in adults, which is associated with a highly disabling condition. To date, ALS remains incurable, and the only drugs approved by the FDA for its treatment confer a limited survival benefit. Recently, SOD1 binding ligand 1 (SBL-1) was shown to inhibit in vitro the oxidation of a critical residue for SOD1 aggregation, which is a central event in ALS-related neurodegeneration. In this work, we investigated the interactions between SOD1 wild-type and its most frequent variants, i.e., A4V (NP_000445.1:p.Ala5Val) and D90A (NP_000445.1:p.Asp91Val), with SBL-1 using molecular dynamics (MD) simulations. The pharmacokinetics and toxicological profile of SBL-1 were also characterized in silico. The MD results suggest that the complex SOD1-SBL-1 remains relatively stable and interacts within a close distance during the simulations. This analysis also suggests that the mechanism of action proposed by SBL-1 and its binding affinity to SOD1 may be preserved upon mutations A4V and D90A. The pharmacokinetics and toxicological assessments suggest that SBL-1 has drug-likeness characteristics with low toxicity. Our findings, therefore, suggested that SBL-1 may be a promising strategy to treat ALS based on an unprecedented mechanism, including for patients with these frequent mutations.

## 1. Introduction

Amyotrophic lateral sclerosis (ALS) is a highly disabling neurodegenerative disorder characterized by the progressive and widespread loss of voluntary motor activity, which results in a devastating condition with an enormous impact on patients, families, caregivers, and medical professionals [1]. ALS presents a rapid progression, usually leading to death within three to four years after symptoms onset due to respiratory failure [2]. ALS is the most frequent motor neuron disorder in adults, affecting up to 5 per 100,000 new individuals each year [3]. Due to population aging, the total number of ALS cases is projected to increase by 69% in the next two decades, especially in developing countries [4]. The mean age of ALS onset is between 58–63 years in sporadic cases, and even younger in familial cases, i.e., between 40–60 years, affecting individuals within the working age [5]. It results in a significant economic burden, estimated at over one billion dollars per year in the United States only, attributed to loss of production, caregiver needs, and medical costs [6]. To date, ALS remains incurable, and only two drugs have been approved by the FDA for its treatment: riluzole and edaravone, which confer a limited survival benefit of a few months. Thus, there is an urgent need to develop more effective treatments for ALS [7].

ALS can be classified as sporadic (sALS) and familial (fALS), which correspond to 90% and 10% of all cases, respectively. Missense mutations in the *SOD1* gene are considered an important cause of ALS, being associated with 23% of all fALS cases, and up to 7% of sALS cases [8]. The protein encoded by the *SOD1* gene, superoxide dismutase 1 (SOD1), is a homodimeric metalloenzyme that catalyzes the dismutation of superoxide anion to hydrogen peroxide, which plays a central role in the endogenous antioxidant defense system [9]. SOD1 is ubiquitously expressed and localizes mainly to the cytosol, representing 1–2% of the total soluble protein in the central nervous system [10]. Conditions such as impaired post-translational modifications, oxidation, and mutations can lead to SOD1 misfolding and toxic gain of function, initiating protein aggregation events that ultimately lead to ALS-related neurodegeneration [11].

Recently, SOD1 binding ligand 1, also known as SBL-1, has shown promising in vitro results in inhibiting the oxidation of Trp32, a critical residue for SOD1 aggregation. SBL-1 is an isoproterenol analog with a significant affinity for SOD1 [12]. Oxidation of the Trp32 side chain is known to potentiate SOD1 aggregation and cytotoxicity [13], which is central to the pathophysiology of ALS [14]. Despite its potential as an anti-ALS drug, no assays on the toxicity and pharmacokinetics properties have yet been performed for the SBL-1 compound [15]. Among the ALS-related mutations in SOD1, A4V (NP_000445.1:p.Ala5Val) and D90A (NP_000445.1:p.Asp91Val) are the most prevalent worldwide, accounting for approximately half of all ALS-SOD1 cases in the United States and Europe, respectively [16]. A previous study by our group indicated that these mutations promote important structural and dynamic alterations in the SOD1 protein [2], which could affect SBL-1 binding modes and affinity, influencing the individual drug response [17].

The safety and efficacy of drug candidates are key factors in drug development [18]. In silico data on drug toxicity and pharmacokinetics are becoming increasingly important, particularly considering the growing number of restrictions imposed on in vivo testing by International Regulatory Agencies [19]. Moreover, differences in the genetic background existing in the overall population significantly influence drug efficiency, which can manifest as limited pharmacological activity [18]. Following the methodology previously established by our group [20,21,22], we investigated in silico the interactions between SOD1 wild-type and its variants A4V, and D90A with the SBL-1 compound, in addition to assessing the ADMET (absorption, distribution, metabolism, excretion, and toxicity) profile of SBL-1.

The predictive analyses we performed suggest that SBL-1 is a low-toxic compound with drug-likeness characteristics. Our findings further indicate that the SBL-1 mechanism of action is possibly preserved upon relatively frequent mutations on its receptor, which implies that SBL-1 could also be promising for the treatment of ALS in patients carrying these mutations. Initial evidence on SBL-1 safety, pharmacokinetics, and pharmacodynamics, such as those provided in this study, could be useful to guide future experiments in vitro and in vivo, saving time and resources [23].

## 2. Materials and Methods

### 2.1. Data Retrieval

The crystallographic structure of human SOD1 wild-type in complex with the SBL-1 compound was obtained from the Protein Data Bank (ID: 5YTO) [24]. Additional information on SBL-1 structure and safety were obtained from the PubChem database (ID: 134828057) [15]. The SOD1-SBL-1 interactions observed in the crystallographic structure 5YTO were designed into a bidimensional diagram using the PoseView software [25] and represented as an equivalent three-dimensional scheme using Pymol software [26].

### 2.2. Molecular Dynamics Simulations

Mutator Plugin 1.3. available on the Visual Molecular Dynamics (VMD) 1.9.3 [27] was used to generate three-dimensional structures of variants A4V and D90A by inducing the respective mutations on the crystallographic structure of wild-type SOD1 (PDB ID: 5YTO). The protonation state of SBL-1 was predicted at pH 7.4 using the ACD/pKa GALAS module (Advanced Chemistry Development. Inc., Toronto, ON, Canada). SBL-1 preparation consisted of adding hydrogens to the molecule, which was performed by the Protoss server [28]. SBL-1 was then submitted to the SwissParam server, generating the topology and parameters compatible with the CHARMM27 force field [29]. The protonation state of SOD1 was predicted using the ProteinPrepare plugin available at the PlayMolecule server, considering a pH of 7.4 [30]. Finally, SOD1 preparation consisted of manually selecting the protonation state of each protein residue inside the GROMACS’s user interface.

Molecular Dynamics (MD) simulations of SOD1 wild-type and variants complexed with SBL-1 were performed in triplicates using the GROMACS 2020.6 package [31], which followed the methodology previously established by our group [20]. The crystallographic structure of 5YTO was used as the starting point of the simulations. CHARMM27 was selected as the force field [32]. The simulations occurred inside a triclinic box, which was solvated by TIP3P water models, neutralized by adding Na^+^ and Cl^−^ ions (0.15 mol/L), and then minimized using the steepest descent method. After the energy minimization step, an NVT ensemble (constant number of molecules, volume, and temperature) was followed by an NPT ensemble (constant number of molecules, pressure, and temperature). NVT was performed at 300 K for 100 ps using a v-rescale thermostat [33]. NPT was performed at 300 K and 1 atm for 100 ps using a Parrinello-Rahman barostat [34] and v-rescale thermostat [33]. Finally, the production simulation was performed at 300 K for 300 ns. The particle mesh Ewald algorithm [35] was used to process electrostatic interactions, and the Linear Constraint Solver algorithm [36] was used to constrain the covalent bonds.

### 2.3. Trajectory Analysis

The trajectories were concatenated and analyzed using the following GROMACS distribution programs: gmx trjcat, gmx trjconv, gmx rms, gmx rmsf, gmx gyrate, gmx sasa, gmx hbond, gmx mindist, gmx distance, and gmx gangle. The SBL-1 atoms and SOD1 backbone atoms were used for the root-mean-square deviation (RMSD). SOD1 atoms were used to calculate the radius of gyration (Rg) and solvent-accessible surface area (SASA). The number of protein-ligand contacts and minimum distances between these groups were assessed using a cut-off range of 5 Å [20].

The most frequent types of protein-ligand interactions were computed over time, i.e., hydrogen bonds, ionic bonds, hydrophobic contacts, π-stacking, and cation-π interactions [37]. In this work, the donor(H)-acceptor distances were considered for the calculation of hydrogen bond interactions. These interactions are commonly considered by algorithms used to create representations of protein-ligand binding, like LigPlot, PoseView [25], and nAPOLI [38]. The hydrogen bonds formed between SOD1 and SBL-1 were assessed based on angles ≤30° and distances ≤3.5 Å [20]. Hydrophobic contacts were computed considering a maximum distance of 4 Å between carbon and sulfur atoms, according to the definition proposed by Alvarez (2013) [39]. The following geometric restrictions were considered for the π-stacking interactions: (i) parallel stacking: acute angle between ring planes ≤22° and distance between ring centroids ≤4.5 Å; (ii) perpendicular stacking: acute angle between ring planes ≥67° and distance between ring centroids ≤6 Å. π-stacking interactions were defined according to the experimental distribution of protein-ligand complexes included in the TOUGH-D1 dataset, as discussed by Brylinski (2018) [40]. Cation-π interactions were defined according to the geometric criteria proposed by Kumar et al. (2018). Kumar et al. (2018) considered a cylinder model in which valid cation-π interactions occurred within a maximum height of 6 Å from the aromatic ring (perpendicular) and a maximum distance of 2.3 Å from the ring centroid along with the horizontal plane [41]. Ionic bonds were computed based on distances ≤4 Å from the center of mass of opposed charged groups, as defined by Piovesan et al. (2016) [42].

VMD 1.9.3 [27] was used to analyze the occupancy of hydrogen bonds formed between SOD1 and SBL-1 during the simulation based on angles ≤30° and distances ≤3.5 Å [20]. Conformational ensembles of the SOD1-SBL-1 complex were extracted from the concatenated trajectories using the software gmx cluster available in the GROMACS 2016.6 package. The Gromos method and an RMSD cut-off of 2.0 Å from the complex atoms were considered for the clustering analysis. Representative structures for the protein-ligand complexes, i.e., wild-type and mutants, were obtained by extracting the centroid structure from the most populated cluster of SOD1-SBL-1 conformations [43]. The representative structures were obtained from the equilibrated portion of the concatenated trajectories. The binding modes observed in the representative structures were assessed using the Pymol [26] and PoseView software [25].

Finallly, the concatenated trajectories were submitted to an MM-PBSA (molecular mechanics Poisson-Boltzmann surface area) analysis, which was performed using g_mmpbsa software [44]. G_mmpbsa requires an old version of a .tpr file containing the molecular system, which is generated by GROMACS 2018.1 or older. The .tpr files generated in the newest versions of GROMACS contain essentially the same technical information as those stored in the old .tpr file, but with a few format changes (only) that affect its compatibility with g_mmpbsa. To manage this limitation, we generated an old .tpr file containing the same molecular system as those simulated in this study but using GROMACS version 2018.1 instead of 2020.6. MMPBSA was used to estimate the binding free energy of the SOD1-SBL-1 complex over time. The energy contribution of each amino acid to SBL-1 binding was also assessed. MMPBSA estimates the binding affinity mainly based on three energetic terms: (i) Molecular Mechanics (MM), attributed to potential energy calculated in a vacuum condition using an MM force field, which includes bonded terms related to interactions between atoms linked by covalent bonds, and nonbonded, or noncovalent, terms that describe the van der Waals forces and electrostatic interactions; (ii) Poisson-Boltzmann (PB) characteristics, attributed to the polar solvation energy, which is estimated using the Poisson-Boltzmann equation considering the repulsion and attraction between solute and solvent molecules; (iii) surface area (SA) attributed to the nonpolar solvation energy, which is calculated based on the assumption that the solvent accessible solvent area is linearly related to the amount of apolar energy [44].

### 2.4. In Silico Pharmacokinetics and Toxicological Assessment

The pharmacokinetics and toxicity profile of the SBL-1 compound was characterized in silico using the ADMET Predictor™ 10.4 (Simulations Plus, Inc., Lancaster, CA, USA). ADMET Predictor™ is a statistical-based QSAR (Quantitative Structure-Activity Relationship) software.

Hepatotoxicity, mutagenicity, carcinogenicity, cardiotoxicity, and acute toxicity were combined into a unique score, the TOX risk. Hepatotoxicity was estimated by assessing the serum levels of liver-damage-related enzymes. Mutagenicity risk (MUT risk) was analyzed using in silico variations of Ames tests, including assays with *Salmonella typhimurium* strains (TA97, TA98, TA100, TA1535, and TA1537), and repair-deficient *Escherichia coli* strains (WP2), with or without metabolic activation [45]. Mutagenicity was also evaluated by using the MUTx risk model, which is a refined version of MUT risk that provides better sensitivity and specificity. Acute rat toxicity was estimated based on the amount of orally administered chemicals required to kill half of the rats tested within 24 h (LD50) [46]. The cardiotoxicity model was based on hERG (Ether-à-go-go Related Gene) potassium channel inhibition [47]. Chronic toxicity was evaluated using rat and mouse-based models considering the oral daily dose administered throughout their lifetimes required to produce tumors in 50 percent of the population (TD50) [48]. Reproductive toxicity was also assessed using the Repro TOX model, which predicts potential adverse effects on sexual organs, teratogenicity, and fertility. Finally, we investigated the drug-drug interaction (DDI) ability of SBL-1 using CYP inhibition models (2C9, 3A4, 1A2, 2D6, and 2C19).

Absorption risk (Absn risk) was assessed to better understand the potential oral absorption problems SBL-1 may have. This score was calculated based on a combination of the following features: size, number of rotatable bonds, hydrogen bond donors, hydrogen bond acceptors, charge, lipophilicity, effective permeability in human jejunum, and water solubility. For further evaluation of drug-like properties, we also used Lipinski’s rule of five [49].

Metabolic risk (CYP risk) was predicted based on the compound’s oxidation by cytochrome P450 (CYP) enzymes. CYP risk was attributed to compounds with elevated intrinsic clearance levels calculated using recombinant assays (CYP1A2, CYP2C9, CYP2C19, CYP2D6, CYP3A4) and a human liver microsomal model.

The overall ADMET risk factor combines TOX risk, Absn risk, CYP risk, and two potential distribution/excretion problems: (i) volume of distribution in humans tissues at the steady-state (Vd), which is proportional to the amount of compound distributed into body tissues in physiological conditions, and (ii) the fraction of unbound compound in human plasma (fu).

The CYP (phase I) and UGT (UDP-glucuronosyltransferase) metabolism (phase II) models available at the ADMET Predictor™ were used to predict the possible phase I and phase II metabolites of SBL-1. Drugs can directly undergo phase I and phase II metabolism or pass through initial phase I modifications and then phase II modifications [50], which were considered in our analysis.

The predicted metabolites underwent toxicity assessment in the ADMET Predictor™ software, which includes mutagenicity, cardiotoxicity, reproductive toxicity, and CYP inhibition. The analysis we carried out did not include hepatotoxicity, carcinogenicity, and acute toxicity, since models were trained based on orally administered compounds.

Due to the complementary nature of the statistical-based and rule-based QSAR methods, we also assessed the ADMET profile of the SBL-1 compound using the ACD/Percepta Platform (Advanced Chemistry Development. Inc., Toronto, ON, Canada).

The Drug Profiler module available in the ACD/Percepta Platform was used for the evaluation of SBL-1 viability. The Drug Profiler module integrates three groups of predictors: PhysChem, ADME, and Drug-safety. Most of Percepta’s models use a probabilistic model combined with a knowledge-based expert system that identifies structural alerts. Experimental evidence on structurally related compounds is also provided by some predictors.

PhysChem performs a drug-likeness evaluation of the compound based on physicochemical features, including lipophilicity (log P), molecular weight, hydrogen bonding (acceptor and donors), solubility, number of rings, and flexibility (rotatable bonds). PhysChem predictor considers some additional cut-offs associated with the concept of lead-likeness.

ADME profiler assesses the pharmacokinetics aspects of the drug candidate. The human intestinal absorption (HIA) model estimates the extent of passive jejunal absorption for orally administered compounds. The Caco-2 (colorectal adenocarcinoma cells) model assesses the compound’s permeability across Caco-2 cell monolayers, considering the transcellular and paracellular routes. The CNS-access (central nervous system) model is based on the blood-brain barrier (BBB) penetration in rodents achieved through passive diffusion. The plasma protein binding (PPB) model estimates the percentage of target compounds bound to human plasma proteins. Fianlly, the metabolic stability model predicts the t^1/2^ of drug candidates in liver microsomes (HLM).

The mutagenicity, cardiotoxicity, and DDI potential of SBL-1 were analyzed using the drug-safety profiler. The metabolites predicted by ADMET Predictor™ for the SBL-1 compound were also evaluated using this module. Mutagenicity was assessed using an Ames model based on the most frequent assays of *S. typhimurium* strains in addition to *E. coli* WP2 uvrA, with or without metabolic activation. Cardiotoxicity was predicted based on an hERG inhibition model considering IC50. DDI was analyzed based on CYP 3A4, 2D6, 2C9, 2C19, and 1A2 inhibition models. The estimated IC50 values were used to classify whether the compound is a CYP inhibitor.

Acute toxicity was additionally investigated for SBL-1 using the Acute Toxicity Hazards module. This model is based on the identification of hazardous fragments (alerts) responsible for high acute toxicity in rodents (Advanced Chemistry Development. Inc., Toronto, ON, Canada).

## 3. Results and Discussion

### 3.1. Data Retrieval

**Figure 1:**
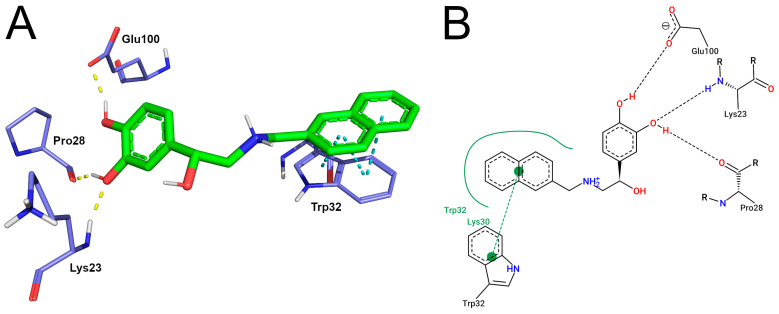
SOD1-SBL-1 binding mode observed in the crystallographic structure 5YTO. (**A**) Three-dimensional representations of SOD1-SBL-1 interactions were designed using the Pymol software. The SBL-1 molecule is represented by green sticks and colored by atom type. The interacting residues of SOD1 are represented by purple lines, colored by atom type, and labeled with the corresponding amino acid residue. Hydrogen bonds are represented by dashed yellow lines, while π-stacking is represented by dashed cyan lines. Residues involved in hydrophobic contact with SBL-1 are also shown. (**B**) Bidimensional diagram of SOD1-SBL-1 interactions designed using the PoseView software. SBL-1 and interacting residues of SOD1 (labeled) are represented by black lines and colored by atom type. Hydrogen bond interactions are represented by dashed black lines, and hydrophobic contacts are represented by continuous green lines.

SBL-1 (4-[(1R)-1-hydroxy-2-(naphthalen-2-ylmethylamino)ethyl]benzene-1,2-diol) is a naphthalene-catechol-linked compound stored at the PubChem database under the identification code: 134828057. SBL-1 is an R-enantiomer with a low molecular weight (309.4 g/mol). To date, no information on pharmacokinetics and toxicity has been computed for the compound [15]. The human SOD1 structure was co-crystallized with SBL-1 and stored under the identification code 5YTO in the Protein Data Bank [24]. 5YTO is a high-resolution structure (1.9 Å) that covers the complete SOD1 length. The binding mode registered in the crystallographic structure, shown in Figure 1, suggests that the catechol portion of SBL-1 forms hydrogen bonding with the residues Glu100, Pro28, and Lys23 of SOD1. The naphthalene moiety of SBL-1 performs π-stacking interactions with the indole group of Trp32, in addition to hydrophobic contacts with Trp32 and Lys30.

Trp32, which is a hydrophobic residue, lies unusually exposed on the beta-barrel surface of SOD1. Trp32 oxidation is a normal modification present in wild-type SOD1 and is also observed in disease-related variants. The study of Taylor et al. (2007) investigated in vivo the effects of Trp32 oxidation on SOD1 aggregation and neuron survival. The study suggested that changing Trp32 to Phe, which has a slower oxidation rate, significantly attenuates cytotoxicity and the formation of inclusions in motor neurons of mouse spinal cords. These results, therefore, indicate that Trp32 oxidation exacerbates SOD1 toxicity and potentiates aggregation [13]. Manjula et al. (2018) performed an in vitro oxidation assay to investigate the SBL-1 ability to inhibit Trp32 oxidation. The study suggested that SBL-1 significantly inhibits the oxidation of this key residue. Interestingly, when the same experiment was performed with the isoproterenol compound, which is an SBL-1 analog without the naphthalene moiety, only weak inhibition on Trp32 oxidation was observed. Based on this information and the interaction profile observed in the crystallographic structure 5YTO (previously shown in Figure 1), Manjula et al. (2018) hypothesized that the protective role of SBL-1 mainly occurs due to the proximity of binding, which might occlude the indole group of Trp32, avoiding oxidation. The authors emphasized the importance of the following interactions for protecting Trp32: (i) π-stacking interactions between the naphthalene moiety of SBL-1 and the indole ring of Trp32; (ii) electrostatic interactions occurring at the dihydroxy phenyl moiety of SBL-1. Hydrophobic interactions may be also important for stabilizing SBL-1 [12].

### 3.2. Molecular Dynamics Simulations

X-ray crystallography, which is among the most used methods for determining macromolecular structures, only provides static snapshots [51]. Crystallography also subjects the protein to conditions of high salinity, which could affect the interaction forces existing in the system, inducing conformations different from those observed in a biological environment [52]. MD simulations, in turn, can accurately reproduce the dynamics of proteins and other molecules in their biological environment. This in silico method generates comprehensive information on protein-ligand interactions and a relatively precise binding affinity estimator, which are relevant to ensure drug efficacy [20,53]. MD is a powerful approach for analyzing drug binding as it allows investigating the entire binding process, including conformational rearrangements in the receptor, alternative binding modes of the ligand, and solvent participation in the interaction [54]. MD also allows the study of perturbations such as mutation and post-translational modifications on protein structures [51], which have proved to be valuable for personalized medicine [55].

A previous study by our group suggested that the mutations A4V and D90A, which account for approximately half of all ALS-SOD1 cases in the US and Europe, lead to a substantial impact on SOD1 structural flexibility and essential dynamics [2]. These alterations, which occur at the proposed biological receptor of SBL-1, can lead to strong and non-intuitive effects on protein binding affinity and specificity [56] with consequent influence on individual drug response [17]. We then performed MD simulations of SOD1 wild-type and variants with SBL-1 to provide additional evidence on the compound’s pharmacodynamics, and to better understand how the pharmacological activity of SBL-1 could be affected by the most frequent missense mutations on its receptor, i.e., A4V and D90A.

The ACD/pKa GALAS prediction suggested that the secondary amine of SBL-1 is protonated at pH 7.4. Notably, the protonation state predicted by SBL-1 using the ACD/pKa GALAS algorithm agreed with the prediction by the Protoss algorithm, which is the standard method to add hydrogens available in the PoseView server (Figure 1). According to Vanni et al. (2011), most β-adrenergic agonists and antagonists have positively charged amine groups, including isoproterenol [57], which is a compound analog to SBL-1 [12]. It thus provides additional evidence on the protonated state of SBL-1 upon physiological pH.

**Figure 2:**
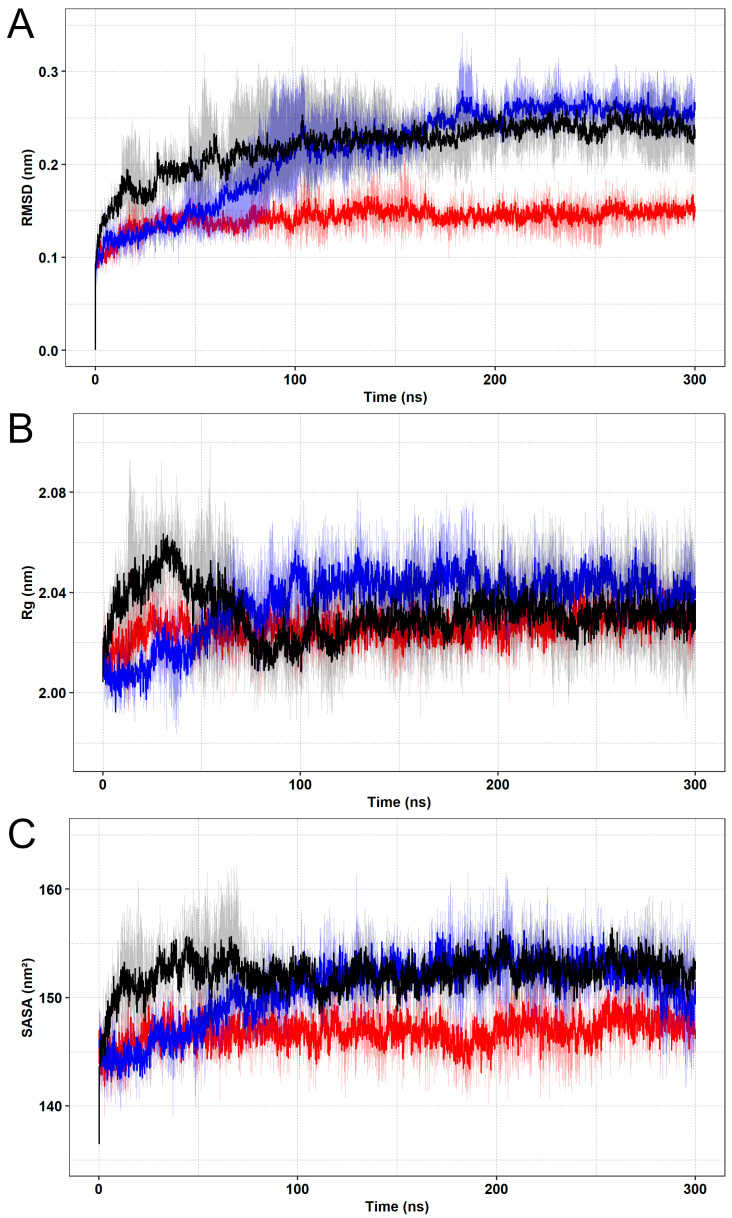
RMSD, Rg, and SASA analyses of the human SOD1 protein. The wild-type is represented in black, variant A4V is represented in red, and variant D90A is represented in blue. The means (solid lines) and confidence intervals (smooth lines) are displayed for the triplicates. (**A**) The RMSD values computed from the backbone atoms of SOD1 are shown as a function of time. (**B**) The Rg values computed from the protein atoms are shown as a function of time. (**C**) The SASA values computed from the protein atoms are shown as a function of time.

RMSD is widely used to measure the atomic displacement between the coordinates of a starting structure and all its subsequent conformations registered over time [58]. This parameter allows for analyzing the time-dependent motions of protein-ligand complexes in a molecular system, in addition to determining its structural convergence throughout the simulation [20]. The RMSD values calculated from the SOD1 wild-type and variants’ conformations are shown in Figure 2A. This analysis indicated that the triplicates presented similar behavior during the simulations when considering their respective means and confidence intervals [2]. An initial moment of structural instability was observed in all trajectories. This might occur due to the initial kinetic shock experienced by molecular systems upon simulation processes [59]. Thus, during this period, variations in structural parameters were not representative of the protein’s behavior. The early simulation effects, i.e., those observed in the first 160ns, were not considered for further analysis aiming at meaningful comparisons. It was followed by the establishment of plateaus in RMSD values for SOD1 wild-type and variants, which remained until the end of all trajectories, indicating that the protein structures fluctuate around average stable conformations. This condition is suggestive of system equilibration, i.e., the period when the physicochemical properties of the molecular system reached an equilibrium state [20]. The simulations of variant A4V were the first to reach equilibrium (≅25 ns), which was followed by the wild-type (≅100 ns), and finally, variant D90A (≅160 ns). Variant A4V (0.15 ± 0.01 nm) presented a lower average RMSD when compared to the wild-type (0.24 ± 0.03 nm), indicating that this variant may deviate less from its starting structure. No differences in average RMSD were registered for wild-type SOD1 and variant D90A (0.26 ± 0.02 nm).

To further assess system equilibration, we performed Rg (Figure 2B) and SASA (Figure 2C) analyses of SOD1 [22]. Rg is a measure of the displacement of all atoms in a protein structure from their common center of mass, providing information on the protein’s overall dimensions or volume [20]. SASA, in turn, is a measure of the exposed surface area in protein structures, which is related to the protein’s ability to interact with solvent molecules, and also proportional to its degree of exposure to the environment [60]. Plateaus of Rg and SASA values were observed after approximately 100 ns in all simulations, which suggests stable protein folding for SOD1 wild-type and variants [61], thus reaffirming system equilibration [22]. The average Rg registered for the wild-type, variant A4V, and variant D90A were, respectively, 2.03 ± 0.02 nm, 2.03 ± 0.01 nm, and 2.04 ± 0.01 nm, while their average SASA values were 152.71 ± 2.20 nm^2^, 147.11 ± 2.30 nm^2^, and 152.48 ± 2.92 nm^2^. Overall, the proteins presented similar Rg and SASA values, except for variant A4V, which presented a lower average SASA (147.11 ± 2.30 nm^2^) when compared to the wild-type (152.71 ± 2.20 nm^2^). This, therefore, indicates that the analyzed mutations may not affect the volume of SOD1, but A4V may reduce the protein’s exposed surface with consequent impact on its surface-to-volume ratio [62].

**Figure 3:**
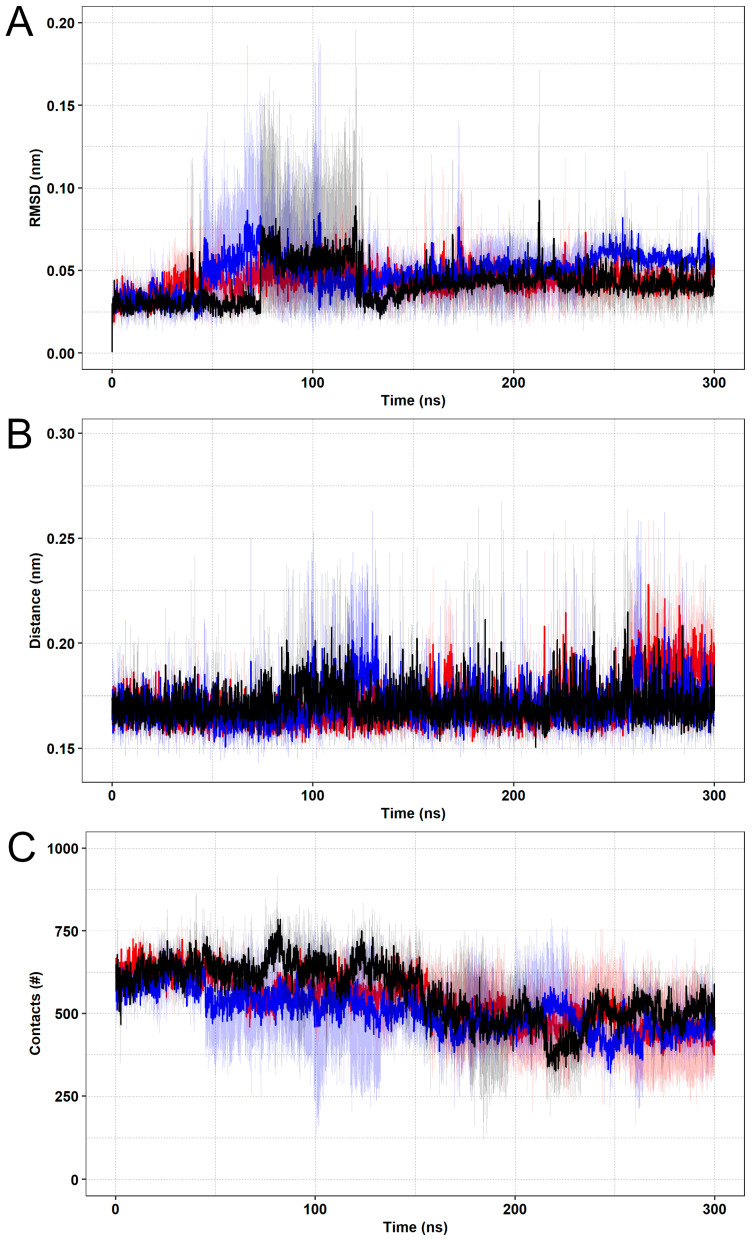
Ligand RMSD, minimum distances, and contact analyses. The wild-type is represented in black, variant A4V is represented in red, and variant D90A is represented in blue. The means (solid lines) and confidence intervals (smooth lines) are displayed for the triplicates. (**A**) The RMSD values computed from the SBL-1 atoms when complexed to SOD1 wild-type are shown as a function of time. (**B**) The minimum distances between SOD1 and SBL-1 are shown as a function of time. (**C**) The number of contacts formed between SOD1 and SBL-1 is shown as a function of time.

The RMSD computed from the SBL-1 atoms is shown in Figure 3A. This analysis shows that the compound presented a steady behavior when complexed to SOD1, given that the RMSD values fluctuated over average values (plateau) after approximately 140 ns in all simulations, which is suggestive of ligand binding stability [20]. No differences in average ligand-RMSD were observed between the wild-type (0.044 ± 0.014 nm) and its variants A4V (0.045 ± 0.011 nm) and D90A (0.054 ± 0.013 nm). Noticeably, the RMSD values computed for SBL-1 remained below 2 Å throughout the simulations, which, according to Liu & Kokubo (2017), further indicates the stability of the ligand binding pose [63].

The minimum distances between SOD1 and SBL-1 computed during the simulations are shown in Figure 3B. This analysis indicates that the wild-type and variants remained at a relatively short and constant distances from SBL-1 during the entire simulation. Similar distances were registered for SOD1 wild-type (0.171 ± 0.014 nm), variant A4V (0.175 ± 0.022 nm), and variant D90A (0.171 ± 0.014 nm). The number of contacts within 5 Å formed between SOD1 and SBL-1 was also assessed (Figure 3C). This parameter is used as an indicator of possible interactions between two groups, usually protein and ligand [64]. As shown in Figure 3C, SBL-1 remained interacting with SOD1 throughout the simulations. No differences were observed in the average number of contacts formed for the wild-type (485.69 ± 96.53) and its variants A4V (474.88 ± 170.76), and D90A (459.82 ± 96.15).

**Figure 4:**
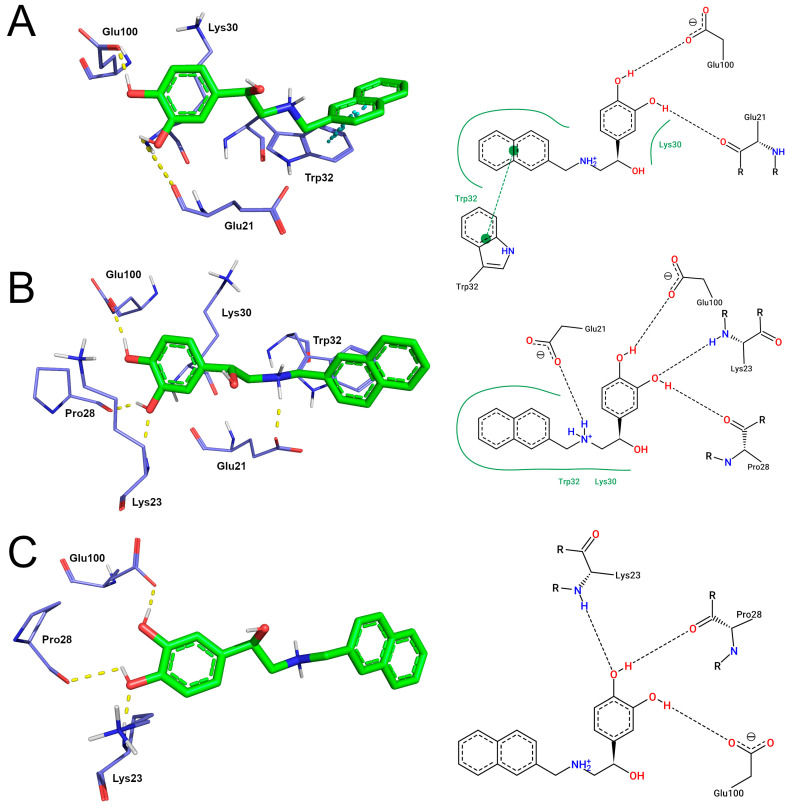
SOD1-SBL-1 binding modes observed in the representative structures extracted from the MD trajectories. Three-dimensional representations of SOD1-SBL-1 interactions were designed using Pymol software. SBL-1 molecule is represented by green sticks and colored by atom type. The interacting residues of SOD1 are represented by purple lines, colored by atom type, and labeled with the corresponding amino acid residue. Hydrogen bonds are represented by dashed yellow lines, while π-stacking is represented by dashed cyan lines. Residues involved in hydrophobic contact with SBL-1 are also shown. The bidimensional diagram of SOD1-SBL-1 interactions was designed using PoseView software. SBL-1 and interacting residues of SOD1 (labeled) are represented by black lines and colored by atom type. Hydrogen bond interactions are represented by dashed black lines, and hydrophobic contacts are represented by continuous green lines. (**A**) Wild-type; (**B**) variant A4V; (**C**) variant D90A.

Clustering is often applied to group protein conformations based on their geometric similarities. In this analysis, protein coordinates extracted from MD trajectories are used to compute the RMSD values within the ensembled conformations. The RMSD values are then used to identify structurally related conformations, which are grouped in the same cluster. The structure with the smallest RMSD between all others inside the cluster, i.e., centroid, is considered the one that best describes the group [20]. When extracted from the most populous cluster, the centroid structure is usually an effective indicator of molecular behavior, given that it represents the largest amount of similar conformations sampled from the MD trajectory [43]. The binding modes registered at the representative structures of SOD1-SBL-1 complexes are shown in Figure 4.

The cluster analysis suggests that the catechol moiety of SBL-1 formed hydrogen bonds with the residues Glu21 and Glu100 of the wild-type. Hydrogen bonds with the catechol moiety of SBL1 were also registered in the crystallographic structure 5YTO (Figure 1), but differed in the number of interactions formed and binding residues (Figure 4A). π-stacking and hydrophobic interactions were also formed between the naphthalene group of SBL-1 and the indole rings of Trp32 in the wild-type (Figure 4A), similar to those observed in 5YTO (Figure 1). An overlay picture of the original (5YTO) and representative conformation of the wild-type SOD1-SBL-1 complex is shown in Figure S1, aiming to show a better visualization of their structural similarities. Moreover, the clustering analysis indicates that three hydrogen bonds were formed between the catechol moiety of SBL-1 and residues Lys23, Pro28, and Glu100 of variant A4V. An additional ionic bond was observed between the secondary amine of SBL-1 and Glu21. Hydrophobic contacts were formed between the naphthalene group of SBL-1 and residues Lys30 and Trp32 of variant A4V. The binding mode registered at the representative structure of variant D90A, in turn, suggesting that the catechol moiety of SBL-1 also formed three hydrogen bonds with Lys23, Pro28, and Glu100. No interactions between SBL-1 and Trp32 were observed for this variant. Notably, the electrostatic interactions with the catechol moiety of SBL-1, emphasized by Manjula et al. (2018) as important for its pharmacological activity, appeared to be preserved in the variants analyzed. On the other hand, the naphthalene interactions with residue Trp32, which are involved in the proposed mechanism of action of SBL-1 [12], may have been affected by mutations.

To further investigate the interaction profile of the SOD1-SBL-1 complex and overcome the limitations of using static structures such as those provided by clustering and X-ray crystallography, we analyzed over time the most frequent interactions involved in protein-ligand binding, i.e., π-stacking, cation-π, hydrogen bonds, hydrophobic contacts, and ionic bonds. These interactions were computed at each simulation frame using the geometric restrictions previously described.

An individual analysis, whose results are shown in Table 1, was carried out to investigate the SBL-1 interactions with Trp32, which are crucial for achieving its proposed pharmacological effects. No ionic or hydrogen bonds were formed between SBL-1 and Trp32 during the trajectories. Thus, they were not included in Table 1. Overall, π-stacking, cation-π, and hydrophobic contacts were formed in a considerable number of frames during the simulations of all proteins (Table 1), which may occlude Trp32, thus protecting it against oxidation. π-stackings and hydrophobic contacts were formed between the indole rings of Trp32 and the naphthalene moiety of SBL-1. Cation-π interactions, in turn, were formed between the indole rings of Trp32 and the charged amine of SBL-1. Among them, π-stacking is believed to be particularly important for this mechanism [12]. The interactions analyzed, including π-stackings, were recorded in a higher number of frames during the simulations of A4V and D90A when compared to the wild-type. To further investigate the SBL-1 ability to occlude the indole rings of Trp32, we assessed the minimum distances registered between these groups. As shown in Table 1, no differences were observed in the average distances from SBL-1 during the simulations of SOD1 wild-type and variants. Our findings, therefore, indicate that SBL-1 may be also effective in protecting Trp32 oxidation upon A4V and D90A variants.

We also assessed the overall interactions of SBL-1-SOD1 complexes, which are shown in Table 2. π-stacking interactions of SBL-1 were only formed with residue Trp32, which was previously discussed individually, thus, not included in Table 2. Hydrogen bonds and hydrophobic contacts were registered in approximately 100% of the trajectory frames. Thus, their analyses are shown numerically rather than as percentages. Hydrogen bonds play a key role in molecular recognition and protein-ligand stability [65]. According to Manjula et al. (2018), the hydrogen bonds formed with the catechol ring of SBL-1 are crucial for stabilizing the complex and might participate in the protection of Trp32 residue [12]. Our analysis suggested that the complexes formed, on average, around two hydrogen bonds during simulations. Moreover, no differences in the average number of hydrogen bonds were observed upon variants when compared to the wild-type. Thus, the mutations analyzed may not impair these interactions (Table 2).

To obtain a deep understanding of how hydrogen bonds are formed, we performed an occupancy analysis, which is shown in Table 3. Hydrogen bond occupancy is defined as the percentage of total conformations registered throughout the simulation in which a ligand performs at least one interaction of this type with a protein residue [66]. Only residues with occupancy equal to or higher than 1% were considered for meaningful comparison, which were Glu21, Lys23, Pro28, and Glu100. Except for Glu21, the three other residues were previously observed as performing hydrogen bonds within the crystallographic structure 5YTO (Figure 1). As shown in Table 3, SOD1 wild-type and variant D90A presented similar hydrogen bonding patterns in which the interactions with Glu100 were the most present, followed by Glu21, then Lys23, and finally Pro28. Variant A4V, in turn, presented a slightly different pattern, with Glu21 as the most frequent residue (71%), followed by Glu100 (51%), then Lys23 (12%), and finally Pro28 (1%).

As described by Manjula et al. (2018), hydrophobic contacts may also be important for stabilizing the SOD1-SBL-1 complex [12]. Our findings suggested that hydrophobic contacts were formed between SBL-1 and residues Lys23, Lys30, and Trp32 in all simulations. The residues formed additional hydrophobic contacts with SBL-1, as follows: (i) wild-type: Glu24, Ser25, Val31, Ser34, and Ile104; (ii) variant A4V: Glu21, Gln22, Val29, Gly33, and Glu100, and (iii) variant D90A: Glu21, Gln22, Pro28, Val29, and Glu100. No differences in the average number of hydrophobic contacts were observed for the variants when compared to the wild-type (Table 2), suggesting that these mutations may not affect the formation of hydrophobic contacts.

Ionic bonding involves electrostatic interactions between ions with opposite charges, which can have a significant influence on the binding strength of biomolecules, including protein-ligand complexes [22,67]. The analysis of ionic bonds suggests that SBL-1 only forms this type of interaction with Glu21. Our findings also suggest that this interaction is frequently formed since it was registered in at least 48% of the simulation frames in all complexes. Furthermore, ionic bonds were considerably more present in the wild-type complex, having been registered during 72% of its trajectories.

Finally, we investigated the cation-π interactions formed within the SOD1-SBL-1 complexes. This analysis suggests that SBL-1 formed three cation-π interactions with SOD1: (i) between the indole rings of Trp32 and the charged amine of SBL-1, as previously discussed; (ii) between the catechol ring of SBL-1 and Lys23, and (iii) between the catechol ring of SBL-1 and Lys30. As shown in Table 2, cation-π interactions were noticeably more frequent during the simulations of variant A4V (55.51%) when compared to the wild-type (19.92%) and variant D90A (13.61%).

Our interaction analysis thus suggested that Glu21, Lys23, Pro28, Lys30, Trp32, and Glu100 may be key residues for stabilizing the SOD1-SBL-1 complex. Overall, variant D90A presented a similar interaction pattern to that of wild-type SOD1, except for the frequency of ionic bonds formed during the simulations, which was considerably lower in the variant. On the other hand, the interactions registered for the A4V complex were notably different when compared to the wild-type, especially considering the ionic bonds and cation-π interactions (Table 2). Despite these differences, A4V formed relatively more hydrophobic and π-stacking interactions with Trp32 (Table 1), which are believed to be crucial for the pharmacological activity of SBL-1 [12].

In a previous study by our group [2], we investigated in silico structural and dynamic alterations in SOD1 protein upon relatively frequent mutations. Among them, A4V and D90A promoted particularly high alterations in the flexibility and essential dynamics of SOD1 functional domains, i.e., electrostatic and metal-binding loops (Figure S2). In the referred study, no flexibility alterations were reported to occur in the corresponding SBL-1 binding site (residues Glu21, Lys23, Pro28, Lys30, Trp32, and Glu21), so it is unlikely to be the main driving factor responsible for the different interaction profiles of SOD1-SBL-1 complexes (Table 1 and Table 2).

**Figure 5:**
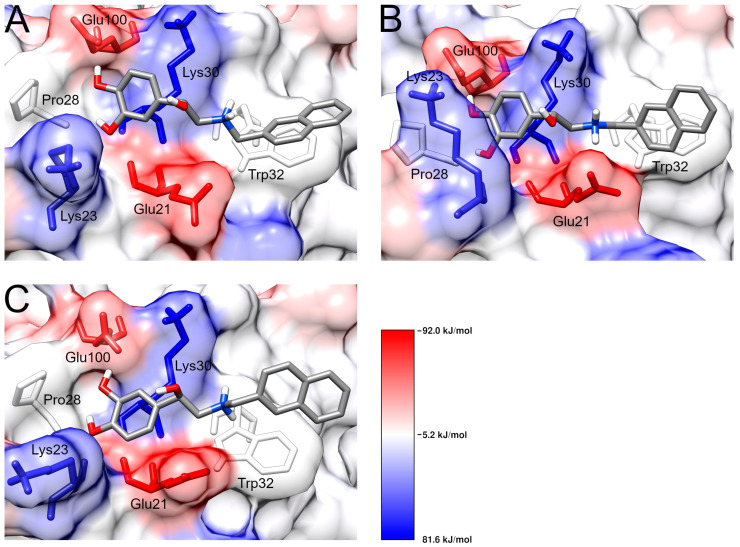
MMPBSA analysis for SBL-1 binding site within SOD1 surface. Residues within the SBL-1 binding site are shown in stick representation and colored according to their energetic contribution, which follows the colorimetric scale displayed in the figure. SOD1 surface is also shown for better visualization. (**A**) Wild-type; (**B**) Variant A4V; (**C**) Variant D90A.

On the other hand, variant A4V presented a reduced SASA when compared to the wild-type (Figure 2), which may affect the SBL-1 binding site, e.g., reducing its size, particularly considering that it is located right in the SOD1 exposed surface (Figure S2). Notably, relatively higher differences in the binding pattern were observed during the simulations of A4V (Table 1 and Table 2). Variant D90A, in turn, presented a considerably more similar interaction profile, and no SASA alterations were observed related to the wild-type. Additionally, as shown in the representative structures extracted from the MD trajectories (Figure 5), variant A4V appears to have a smaller binding site compared to the wild-type, further suggesting that it may have a role in the different binding patterns registered during the simulations. Despite being far from the mutated sites (Figure S2), the amino acid substitutions A4V and D90A may exert long-range effects on binding cavities, influencing protein binding [68].

Binding affinity can be defined as the free energy alteration caused by the formation of a molecular complex. This parameter is useful to measure the strength of protein-ligand interactions, thus being an important initial indicator of drug potency [69]. Negative free energy values indicate favorable processes, i.e., those formed spontaneously, while positive values indicate unfavorable ones [70].

An MMPBSA analysis was then performed on the MD trajectories to estimate the binding affinity of the SOD1-SBL-1 complexes. This method provides an accurate estimation of the binding free energy in protein-ligand complexes, circumventing molecular docking approaches. When combined with molecular dynamics, which provides a sampling of different binding modes, MMPBSA calculations consider the effects of conformational fluctuations and binding entropy, which considerably enhance its predictive power, and greatly increase its computational cost [53]. The binding free energy can be further decomposed to account for the contribution of each protein residue to the interaction [44].

The energetic terms calculated for the SOD1-SBL-1 complexes are shown in Table 4. Overall, the polar solvation term is unfavorable for complex formation, while apolar solvation and MM energies are favorable. MM term dominates the overall binding affinity in all complexes. The binding energy estimated for the compounds further suggests that the formation of the SOD1-SBL-1 complex is a favorable and spontaneous process, reaffirming its viability (Table 4). No differences were observed in the individual energetic terms for the complexes analyzed. Moreover, the total binding energy estimated for the wild-type during the simulations (−129 ± 42 kJ/mol) was similar to that of A4V (−209 ± 52 kJ/mol) and D90A (−146 ± 39 kJ/mol). Despite the different patterns registered for the variants in the interaction analysis (Table 2), our findings indicated that SBL-1 may also bind with significant affinity upon these mutations.

The binding energies computed over time for the SOD1-SBL-1 complexes are shown in Figure S3. The wild-type and variant D90A displayed similar and steady behaviors throughout the simulations, which further suggest complex stability. Variant A4V, in turn, had a relatively less stable behavior but presented numerically lower binding energy values, and thus more favorable ones.

Finally, we investigated the energetic contribution of each residue for complex formation. As shown in Figure S3, the wild-type and variants presented considerably similar contribution patterns, except for position 90 in variant D90A, having an Ala instead of an Asp. Mutation D90A results in the substitution of a negatively charged and hydrophilic amino acid for a non-charged and hydrophobic one, which may be related to the altered binding energy values registered in the referred position (Figure S3). The energetic contribution of Trp32 to the complex formation was not impaired upon A4V and D90A mutations (Table S1), further suggesting that the SBL-1 interactions with this residue, which are involved in its proposed mechanism of action, may be preserved. This analysis also indicates that Glu21, Glu24, Asp96, and Glu100 are the residues that most favor SBL-1 interaction with SOD1, while Lys3, Lys23, and Lys30 are the most unfavored ones. Interestingly, most of these residues were located at the SBL-1 binding site (Figure 5) or nearby regions. Overall, glutamic acid and aspartic acid, which are negatively charged, significantly favor SBL-1 interactions with SOD1. On the other hand, positively charged residues, i.e., histidine, lysine, and arginine, most disfavored complex formation (Table S1).

Amino acids with electrically charged side chains have a deep influence on protein binding affinity to charged ligands such as SBL-1. Interactions between electric charges also confer binding specificity, given that opposite charges attract themselves, whereas like charges repel. Despite being inversely proportional to distance, the strength of charge interactions can be strong even at relatively long ranges, i.e., 5–10 Å [71,72]. This may be related to the energetic contribution pattern previously described for SOD1-SBL-1 complexes from the MMPBSA analysis, in which charged residues located at the SBL-1 binding site (Figure 5) or nearby regions most favored or disfavored the interaction.

Based on the geometric definitions included in the study, cation-π interactions with SBL-1 were formed with residues Lys23 and Lys30 during part of the simulations (Table 2). On the other hand, charged amino acids also exert profound effects on binding to charged ligands due to charge-charge interactions [71]. Thus, a possible explanation for the unfavorable contribution of Lysines to SBL-1 interaction, as suggested by the MMPBSA analysis, is that the repulsive forces between like charges, i.e., charged Lys and protonated amine of SBL-1, would overcome the attractive cation-π forces. Repulsive forces between like-charges can be particularly strong at low ranges [72]. Lys3 remains relatively far from the cut-off range of cation-π interactions, but could still exert long-range repulsive effects through charge-charge interactions [71].

Our in-depth characterization of SOD1-SBL-1 interactions using MD simulations indicates that the mechanism of action proposed by SBL-1 and its binding affinity to SOD1 may be preserved upon relatively frequent mutations on its receptor, i.e., A4V and D90A. Thus, SBL-1 could be promising for the treatment of ALS in patients carrying these mutations. Nonetheless, missense mutations resulting in substitutions to positively charged amino acids, especially those occurring at the SBL-1 binding site or nearby regions, may disfavor the interaction with a consequent impact on SBL-1 activity.

### 3.3. In Silico Pharmacokinetics and Toxicological Assessment

Unsatisfactory ADMET properties are a major cause of drug candidate failure during drug development [73]. Accurate in silico tools for ADMET prediction, such as ADMET Predictor™ and ACD/Percepta, can help decision-making in the early phases of drug discovery, particularly by identifying compounds with the best potential to fit the desired pharmacological properties later in vivo, i.e., those reaching effective concentrations at their targets with low toxicity [74].

The overall evaluation provided by ADMET Predictor™ combines toxicity (TOX risk), absorption (Absn risk), distribution (fu, Vd), and metabolism (CYP risk) issues. Ninety percent of the 2260 commercial drugs available in a reference dataset derived from the World Drug Index (WDI) [75] were estimated to have ADMET risk values below 7. The SBL-1 compound presented only three violations in the overall evaluation: mutagenicity (MUT), hydrogen bond donor (HBD), and high CYP2D6 clearance (2D6), totalizing 2.5 penalties (Table 5), far below the reprobation cut-off (seven penalties).

SBL-1 was only classified as mutagenic by the Ames models of NIHS and *S. typhimurium* TA97 + TA1537 strains without microsomal activation (S). SBL-1 presented a Mut risk of 1.2, thus raising a mutagenic alert (Mut risk ≤ 1.0), but it is still within 90% of the empirical distribution calculated from commercial compounds available in the WDI subset. To further assess mutagenicity, we evaluated the Mutx risk of SBL-1, which is an improved version of Mut risk that provides better sensitivity and specificity within the same cut-off (risk ≤ 1.0) (Simulations Plus, Inc., Lancaster, CA, USA). SBL-1, in turn, presented a Mutx risk of 0.64 (Table 5). Thus, it was not considered mutagenic by the Mutx risk model.

The toxicity analysis suggests that SBL-1 was not classified as hepatotoxic, carcinogenic, cardiotoxic, or acutely toxic, thus receiving a Tox risk penalty of 1, attributed to the mutagenicity in Ames tests, as previously discussed for Mut risk. Ninety percent of the compounds available in the WDI subset presented Tox risk values ≤2.0 (Table 5). Thus, the Tox risk score estimated for SBL-1 was within the expected range for commercial drugs, indicating that SBL-1 could be safe.

Disturbance in critical events in prenatal and early postnatal development by teratogen agents can lead to irreversible defects or dysfunctions [76]. The Repro Tox model, which accounts for reproductive and developmental toxicity, suggested that SBL-1 is non-teratogenic (Table S2).

The estimated Absn risk of SBL-1 was 0.5, which was attributed to undesired hydrogen bond donor properties (HBD). Half of the penalty was attributed to SBL-1 given that it presented an HDB value within the soft threshold, i.e., the region in which the compound violates the cut-off but is still very close to it. Ninety-one percent of the commercial drugs available in the WDI subset have Absn risk values lower than 4 (Table 5). Furthermore, no violations of Lipinski’s rule of five were registered for SBL-1. The rule states that a compound is likely to exhibit poor oral absorption or permeability when ≥2 rules are violated, which applies to most commercial drugs suitable for oral dosing [77]. Given the respective Absn risk and Lipinski’s score values, SBL-1 presented drug-like properties, thus being possibly suitable for oral administration.

Vd and fu models account for the distribution and excretion properties in the ADMET Predictor™ method, which are based on the extent to which a drug is bound in the circulation (fu) or tissues (Vd). According to an analysis from the WDI subset, compounds with Vd < 4 and fu > 4 are likely to have distribution and excretion problems. SBL-1 had a Vd of 16.4 and fu of 2.2, presenting favorable distribution and excretion properties.

CYP risk accounts for possible metabolization issues a compound may have, penalizing elevated levels of intrinsic clearance by the corresponding CYP enzyme. Extensive metabolism with consequently high clearance reduces the half-life and bioavailability of potential drugs, which could compromise their activity in the target organ [78]. SBL-1 was predicted to have a CYP risk of 1 (Table 5), which was attributed to increased intrinsic clearance by CYP2D6. Despite this potential problem, SBL-1 still presents a CYP risk comparable to most commercial drugs (CYP risk ≤ 2), and it may not affect its activity.

The pharmacological treatment of ALS involves the simultaneous administration of multiple drugs, which include the disease-modifying drugs Riluzole and Edaravone, in addition to a plethora of drugs aiming to manage the disease’s symptoms [79]. Thus, we investigate potential DDI issues by evaluating the CYP inhibition ability of SBL-1. Inhibition of cytochrome P450 enzymes is a central mechanism in DDI, which may decrease the metabolization of co-administered drugs [80], leading to adverse drug reactions [81]. CYP inhibition is observed in the majority of commercial drugs, especially CYP3A4 [80,82]. Our findings indicate that SBL-1 may inhibit the activity of CYP1A2, CYP2D6, and CYP3A4 enzymes (Table 5), thus requiring some caution when co-administered with drugs used to treat ALS, particularly SSRIs [83], benzodiazepines [84], and hypnotics [85,86], which are mainly metabolized by CYP2D6, CYP3A4, and CYP3A4, respectively.

Phase I and Phase II modifications produce more polar molecules, facilitating elimination [87]. The metabolism model available in ADMET Predictor™ considered the most frequent reactions within phase I and phase II, i.e., oxidation (CYP model) and glucuronidation (UGT model).

**Figure 6:**
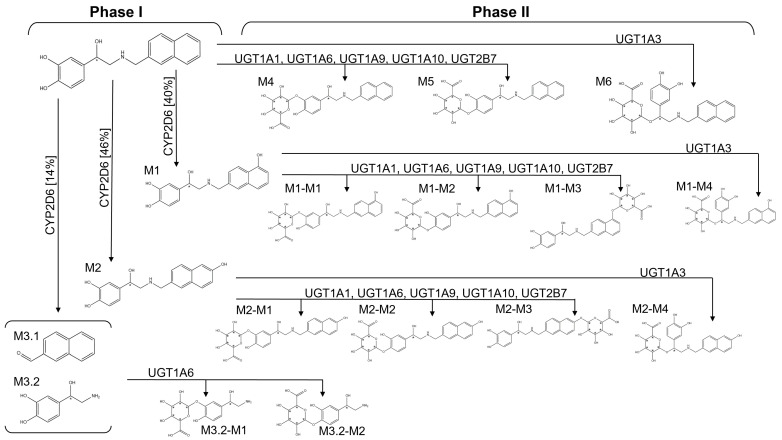
Phase I and phase II metabolism of the SBL-1 compound as predicted by ADMET Predictor™. SBL-1 and its metabolites are displayed as bidimensional chemical structures with their corresponding nomenclature. Each metabolism step is represented by an arrow containing related enzyme(s). The percentage of each metabolite generated during phase I metabolism is shown between brackets. ADMET Predictor™ does not provide the percentage of metabolites generated during phase II. Chiral centers are highlighted by asterisks.

Seventeen metabolites were predicted by ADMET Predictor™ for SBL-1, as shown in Figure 6. Four metabolites result from direct phase I metabolism: M1, M2, M3.1, and M3.2. CYP2D6 was the unique CYP enzyme predicted to metabolize SBL-1 during phase I. M1 was produced from the aromatic hydroxylation of the ortho position at the naphthalene ring, while M2 results from the same modification but at the meta position. M1 and M2 are produced in high levels, corresponding to 86% of all metabolites generated during this phase. M3.1 and M3.2 result from the N-dealkylation of the amine group, which is considered relatively rare, corresponding to only 14% of phase I metabolites. M4, M5, and M6 were directly generated during phase II metabolism. M4 and M5 were produced by the action of UGT1A1, UGT1A6, UGT1A9, UGT1A10, and UGT2B7, which results in the conjugation of a glucuronic acid into the dihydroxy phenyl moiety. M6 was generated through glucuronidation at the hydroxyl group bound to asymmetric carbon, which was catalyzed by UGT1A3. ADMET Predictor™ does not provide the percentage of metabolites generated during phase II.

Additionally, the metabolites M1-M1 and M1-M2 were generated from the subsequent metabolism of M1 by UGT1A1, UGT1A6, UGT1A9, UGT1A10, and UGT2B7, which conjugate a glucuronic acid into the dihydroxy phenyl moiety. M2-M1 and M2-M2 result from a similar process but within the M2 metabolite. M1-M3 and M2-M3 metabolites were formed through the glucuronidation of the ortho and meta hydroxyl moieties present in the corresponding naphthalene rings of M1 and M2. This process was also catalyzed by enzymes UGT1A1, UGT1A6, UGT1A9, UGT1A10, and UGT2B7. M1-M4 and M2-M4 were formed by UGT1A3 upon the glucuronidation of the asymmetric carbon of M1 and M2. Finally, M3.2-M1 and M3.2-M2 metabolites were generated by UGT1A6 through the conjugation of a glucuronide acid into the dihydroxy phenyl moiety of M3.2.

The predicted metabolites of SBL-1 were assessed for potential toxicity hazards, as shown in Table S2. CYP inhibition was also investigated for them, given that some drugs that reversibly inhibit CYP enzymes have circulating metabolites that are CYP inhibitors [88]. The ADMET Predictor^TM^ analysis raised mutagenicity alerts for the metabolites, attributed to positive Ames tests within the NIHS model and *S. typhimurium* TA97 + TA1537 strains without microsomal activation. The maximum Mut risk calculated for the metabolites was 1.2, similar to that described for SBL-1. The Mutx risk analysis indicated that the metabolites presented a maximum of 0.64 Mutx risk score, which is far below the cut-off of 1.0 expected for commercial drugs. No cardiotoxicity was observed for the metabolites, but some of them were considered out-of-scope by the hERG model available in ADMET Predictor™, thus receiving an hERG-tag. The out-of-scope condition was observed in all phase II metabolites, which may be related to the acid glucuronic conjugated to the molecule (Simulations Plus, Inc., Lancaster, CA, USA). According to the safety and hazard profile raised in PubChem, glucuronic acid (IDs: 94715) is not considered cardiotoxic, but it may cause respiratory irritation [15]. Furthermore, no developmental toxicity was observed for the SBL-1 metabolites (Table S2).

ACD/Percepta Platform was further used to characterize the ADMET profile of SBL-1 (Table 6). Combining different QSAR methods, particularly those with complementary nature like ADMET Predictor™ and ACD/Percepta, add robustness to the prediction [23], which could benefit our assessment by providing more solid initial evidence on SBL-1 safety and pharmacokinetics.

The evaluation provided by the PhysChem profiling module indicates that SBL-1 does not violate any of Lipinski’s nor Lead-like rules, i.e., −2 ≤ logP < 4.2, molecular weight < 460, number of hydrogen bond donors ≤ 5, and number of hydrogen bond acceptors ≤ 9. The Lead-like rule is an adapted and stricter form of Lipinski’s rule designed to deal with possible modifications a lead compound would have during its development stage (Advanced Chemistry Development. Inc., Toronto, ON, Canada). The solubility estimated for SBL-1 was 33.9 mg/mL, thus being classified as a soluble compound, which is crucial for a drug to achieve proper concentration in the systemic circulation and further exert its pharmacological response [89]. Based on the analyses of marketed drugs, relatively rigid compounds with few rings are usually more suitable for oral dosing [90,91]. SBL-1 presented 5 rotatable bonds and three rings, which are within the desired range of values adopted by ACD/Percepta, i.e., number of rings ≤ 4 and number of rotatable bonds ≤ 10 (Table 6). Our findings further suggest that SBL-1 is likely to exhibit satisfactory oral absorption and permeability [77].

The assessment by the ADME profiling module provided a model to directly account for bioavailability SBL-1, which was predicted to have 96% bioavailability, thus it would successfully reach the systemic circulation. Additionally, SBL-1 was predicted to have high permeability through Caco-2 scales (16 × 10^−6^ cm/s) and high passive jejunal absorption (HIA: 100%) (Table 6), corroborating results concerning bioavailability previously discussed.

The pharmacological target of SBL-1, i.e., SOD1, is mainly located in the CNS [10]. BBB penetration is crucial, especially for CNS-acting drugs, which must cross the BBB to interact with their target receptors [92]. The CNS access model available in ADME profiling combines descriptors for passive brain penetration (Log BB and Log PS) and the fraction unbound in the brain (fu, brain) to evaluate whether the compound would exhibit CNS activity (Advanced Chemistry Development. Inc., Toronto, ON, Canada). The final CNS access score estimated for SBL-1 was −2.93, suggesting that it would exhibit CNS activity, i.e., score > −3 (Table 6).

ADME profiling module uses a PPB model to account for the compound’s distribution. The free-drug hypothesis states that only unbound drugs are available for interacting with their pharmacological targets [93]. On the other hand, weak PPB makes the drug more available for metabolism and excretion, ultimately reducing its half-life [94]. Thus, the PPB model considers moderately bound plasma proteins, i.e., 40% < PPB ≤ 80%, as the optimal condition for drug candidates. SBL-1 was predicted to have 81% of PPB, which is very close to the upper range of optimal values (Table 6). Thus, it may still present favorable distribution.

As previously discussed, readily metabolized compounds, i.e., those with low t^1/2^ values in HLM, often possess reduced bioavailability and limited activity [95]. ADME profiling classifies compounds with t^1/2^ in HLM < 30 min as metabolically unstable. The classification is based on the HLM score estimated for the compound, which is a combination of the reliability index (RI) and predicted probability (p). The resulting score penalizes probabilities close to 0.5 and low RI, which are indicators of inconclusive prediction. The HLM score predicted for SBL-1 was 0.49, suggesting undefined metabolic stability (Table 6), i.e., 0.33 < HLM score ≤ 0.67.

A similar score system is also used by the Drug-safety profiler module to account for mutagenicity and cardiotoxicity. Additionally, experimental evidence on structurally related compounds is also provided by the predictors included in this module. The mutagenicity model is based on Ames tests. SBL-1 received an Ames score of 0.35, thus being classified as undefined mutagenic, but still very close to the upper range of the non-mutagenic class (Ames score < 0.33), and very far from the bottom range of the mutagenic class (Ames score ≤ 0.67). Additionally, experimental evidence indicates that compounds structurally related to SBL-1, including its analog isoproterenol, are non-mutagenic (Table S3). Thus, together with Mutx risk analysis (Table S2), the Ames model available in the Drug-safety profiler further suggests that SBL-1 may not be mutagenic. In turn, the cardiotoxicity model ACD/Percepta is based on the inhibition of hERG channels. The hERG score estimated for SBL-1 was 0.31, indicating non-cardiotoxicity, which is in agreement with the ADMET Predictor™ evaluation (Table S2). Experimental evidence on compounds structurally related to SBL-1, which includes its analog isoproterenol, further suggests non-mutagenicity, in accordance with data from the literature (Table S3).

The Drug-safety profiler evaluation on CYP inhibition suggests that SBL-1 does not inhibit CYP2C9, but it was inconclusive for CYP3A4, CYP2D6, CYP2C19, and CYP1A2 (Table S4). Notably, the highest CYP scores, i.e., those closer to the inhibitor class, were achieved by CYP3A4, CYP2D6, and CYP1A2 enzymes, which had previously been classified as inhibited by SBL-1 within the ADMET Predictor™ assessment (Table 6). Compounds structurally related to SBL-1, including catecholamines and β1 adrenergic agonists, have not demonstrated CYP inhibition experimentally (Table S4).

No hazardous fragment associated with severe acute toxicity was detected for SBL-1 using the expert system model included in the Acute Toxicity Hazards module. This model accounts for 86 predefined toxicophores compiled from the toxicological literature, i.e., structural fragments that are statistically related to toxic properties in drugs (Table 6) [96].

Finally, the metabolites predicted by ADMET Predictor™ for the SBL-1 compound, as shown in Figure 6, underwent toxicity assessment using the Drug-safety profiler module. No mutagenicity, cardiotoxicity, or CYP inhibition were identified for the metabolites (Table S3).

Overall, the in silico pharmacokinetics and toxicological assessments we carried out suggest that SBL-1 has drug-likeness characteristics with low toxicity, which may be favorable for the development of an anti-ALS compound [74].

## 4. Conclusions

Our MD findings suggest that the complex SOD1-SBL-1 remains relatively stable and interacts within a close distance during the simulations. The MMPBSA analysis indicates that the SOD1-SBL-1 complex formation is energetically favorable, thus occurring spontaneously. Our in-depth characterization of SOD1-SBL-1 interactions suggests that the mechanism of action proposed by SBL-1 and its binding affinity to SOD1 may be preserved upon the relatively frequent mutations A4V and D90A. Thus, SBL-1 could also be promising to treat ALS in patients carrying these mutations. On the other hand, missense mutations resulting in substitutions to positively charged amino acids, particularly those occurring at the SBL-1 binding site or nearby regions, may disfavor the interaction with a consequent impact on its pharmacological activity. The in silico pharmacokinetics and toxicological assessments we carried out suggest that SBL-1 has drug-likeness characteristics with low toxicity, which may be favorable for the development of an anti-ALS compound based on an unprecedented mechanism. Initial evidence on SBL-1 safety, pharmacokinetics, and pharmacodynamics, such as those provided in this work, could be useful to guide future studies in vitro and in vivo, saving time and resources.

## Figures and Tables

**Table 1:** Interactions between SBL-1 and residue Trp32.

Protein	π-Stacking ^1^	Cation-π ^1^	Hydrophobic Contacts ^1^	Minimum Distance ^2^
wild-type	9.02%	3.8%	36.13%	0.37 ± 0.10 nm
A4V	16.88%	38.46%	89.55%	0.26 ± 0.03 nm
D90A	15.76%	10.12%	49.25%	0.34 ± 0.08 nm

^1^ Percentage of frames in which at least one interaction was registered during the trajectories. ^2^ Means and standard deviations are shown.

**Table 2:** Interactions between SBL-1 and SOD1 protein.

Protein	Hydrogen Bonds ^1^	Hydrophobic Contacts ^1^	Ionic Bonds ^2^	Cation-π ^2^
WT	2.42 ± 1.07	10.07 ± 5.69	72.72%	19.92%
A4V	1.98 ± 1.25	11.81 ± 7.63	57.34%	55.51%
D90A	1.78 ± 0.91	8.91 ± 5.88	48.39%	13.61%

^1^ Means and standard deviations are shown. ^2^ Percentage of frames in which at least one interaction was registered during the trajectories.

**Table 3:** Hydrogen bond occupancy of the SBL-1-SOD1 complex.

Protein	Lys23	Glu21	Pro28	Glu100
WT	18.23%	46.76%	5.21%	93.67%
A4V	12.15%	71.51%	1.38%	51.09%
D90A	18.83%	35.74%	6.85%	70.99%

**Table 4:** MMPBSA analysis for the SOD1-SBL-1 complexes.

Protein	ΔE Polar (kJ/mol)	ΔE Apolar (kJ/mol)	ΔE MM (kJ/mol)	Binding Energy (kJ/mol)
WT	229 ± 35	−9.41 ± 1.27	−349 ± 65	−129 ± 42
A4V	205 ± 85	−8.80 ± 2.27	−409 ± 118	−209 ± 52
D90A	240 ± 31	−9.22 ± 1.20	−377 ± 61	−146 ± 39

The respective means and standard deviations are shown.

**Table 5:** ADMET Predictor™ analysis for the SBL-1 compound.

Model	ADMET Risk	Absn Risk	CYP Risk	Tox Risk	MutRisk	Mutx Risk	Vd & Fu	Lipinski’s Rule	Repro Tox	CYP Inhibition
Penalty	2.5	0.5	1	1	1.2	0.64	0	0	_	_
Violation	HBD; MUT; 2D6	HBD	2D6	MUT	S_97; NIHS	S_97; NIHS	No	No	No	CYP1A2, CYP2D6, CYP3A4
Cut-off	≤7	≤4	≤2	≤2	≤1	≤1	_	≤2	_	_

HBD: hydrogen bond donor; MUT: mutagenicity in Mut Risk model; 2D6: increased intrinsic clearance for CYP2D6; S_97: mutagenic in *S. typhimurium* TA97 + TA1537 strains without microsomal activation; NIHS: classified as mutagenic by a model trained on the NIHS’s Ames test dataset;Vd: volume of distribution; Fu: fraction unbound. The underline represents parameters not-applicable to the model. Cut-off based on an empirical distribution calculated from commercial compounds available in the WDI subset.

**Table 6:** ACD/Percepta analysis for the SBL-1 compound.

Module	Model	Parameter Value	Classification	Reference Value
DrugSafetyprofiling	Mutagenicity	0.35	Undefined	≤0.33
	hERG inhibition	0.31	Non-inhibitor	≤0.33
	CYP inhibition	0.33–0.64	Undefined (3A4, 2D6, 2C19, 1A2); Non-inhibitor (2C9)	≤0.33
	Acute Toxicity	_	No hazardous fragment	No hazardous fragment
PhysChem profiling	Lipinski’s Rule	0 violations	Optimal	_
	Lead-like Rule	0 violations	Optimal	_
	Solubility	33.9 mg/mL	Soluble	≥10 mg/mL
	Rotatable bonds	5	Optimal	≤10
	Nº of rings	3	Optimal	≤4
ADME profiling	Bioavailability	96%	High	≥70%
	Caco-2	16 × 10^−6^ cm/s	High	≥7 × 10^−6^ cm/s
	HIA	100%	High	≥70%
	PPB	81%	High	≥40% & ≤80%
	Metabolic Stability	0.49	Undefined	≤0.33
	CNS-Access	−2.93	Sufficient for CNS activity	>3

The underline represents parameters not-applicable to the model. Reference values based on the ACD/Percepta evaluations.

## Data Availability

All relevant data are within the manuscript and Supplementary Materials.

## Supplementary Materials

The following supporting information can be downloaded at: https://www.mdpi.com/article/10.3390/pharmaceutics15041095/s1, Table S1: Energetic contribution of each SOD1 residue to SBL-1 interaction computed from the MMPBSA analysis, Table S2: ADMET Predictor™ analysis of SBL-1 compound and predicted metabolites, Table S3: Percepta analysis of SBL-1 compound and predicted metabolites, Table S4: CYP inhibition analysis of SBL-1 compound and predicted metabolites performed using the ACD/Percepta platform, Figure S1: Overlay picture of the original (5YTO) and representative conformation of the wild-type SOD1-SBL-1 complex extracted from the molecular dynamics, Figure S2: Three-dimensional representation of SOD1-SBL1 complex showing important protein sites, Figure S3: MMPBSA analysis of SOD1-SBL-1 complexes.
