# Supplementary material for: In Silico Analyses of a Promising Drug Candidate for the Treatment of Amyotrophic Lateral Sclerosis Targeting Superoxide Dismutase I Protein
Source: Pharmaceutics. 2023 Mar 29;15(4):1095. doi: 10.3390/pharmaceutics15041095 (PMC10143751; doi:10.3390/pharmaceutics15041095)
Supplement: Supplementary file 1 [file pharmaceutics-15-01095-s001.zip › Figure_S1.pdf]

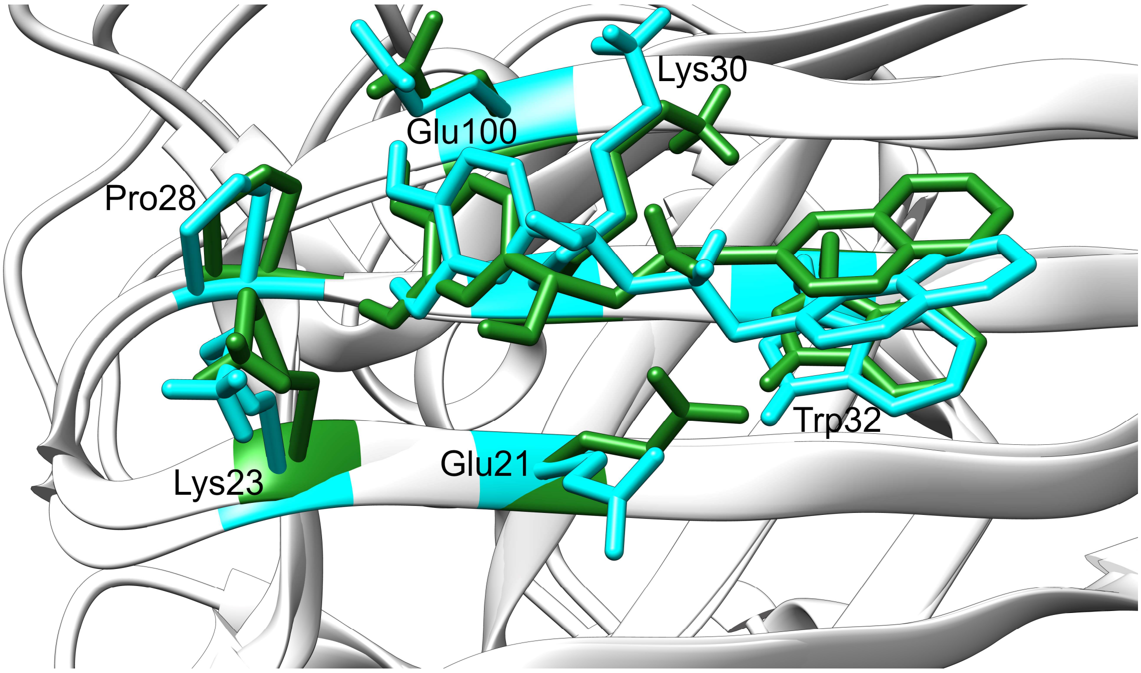

Figure S1. Overlay picture of the original (5YTO) and representative conformation of the wild-type SOD1-SBL-1 complex extracted from the molecular dynamics. The 5YTO conformation is colored green, while the representative conformation is colored cyan. The interacting residues of SOD1 are displayed and labeled with the corresponding amino acid position.
