# Supplementary material for: In Silico Analyses of a Promising Drug Candidate for the Treatment of Amyotrophic Lateral Sclerosis Targeting Superoxide Dismutase I Protein
Source: Pharmaceutics. 2023 Mar 29;15(4):1095. doi: 10.3390/pharmaceutics15041095 (PMC10143751; doi:10.3390/pharmaceutics15041095)
Supplement: Supplementary file 1 [file pharmaceutics-15-01095-s001.zip › Figure_S2.pdf]

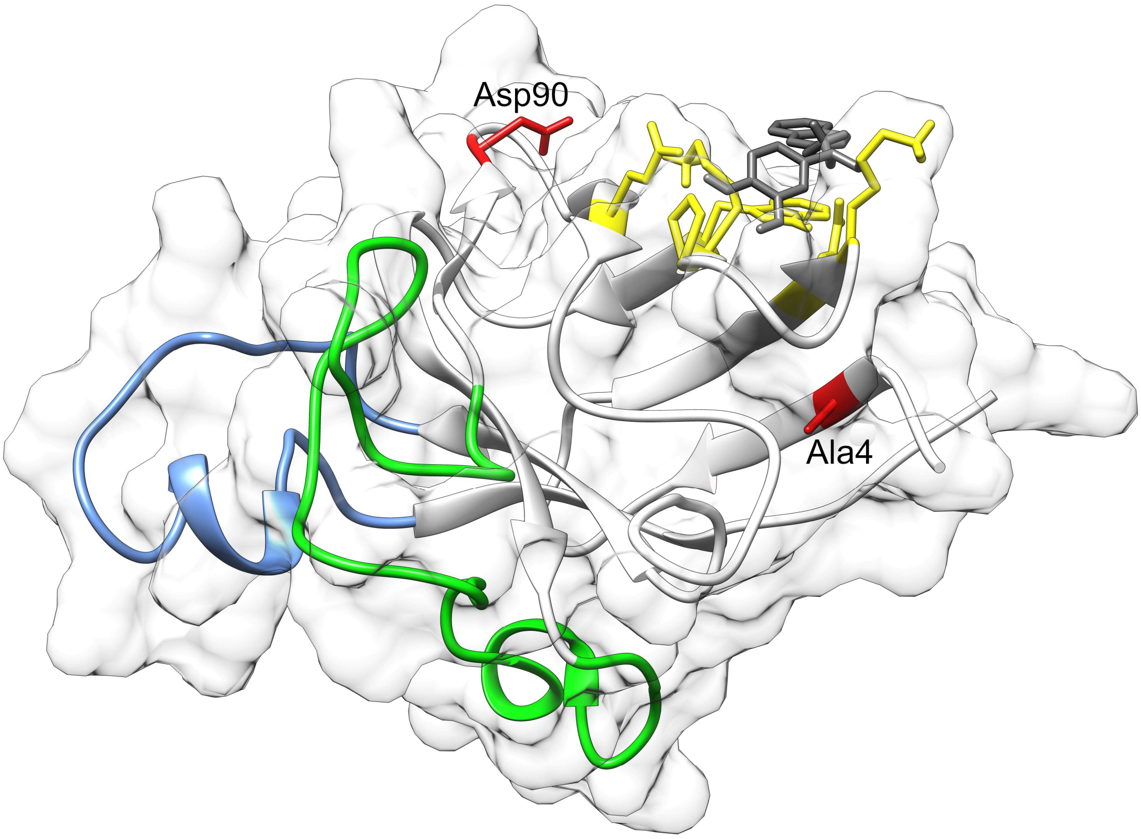


**Figure S2.** Three-dimensional representation of SOD1-SBL1 complex showing important protein sites. The metal-binding and electrostatic loops are colored green and blue, respectively. The amino acid positions affected by A4V and D90A mutations were highlighted in red and labeled accordingly. Residues within the SBL-1 binding site are shown in stick representation and colored yellow, while the SBL-1 compound is represented by dark gray sticks.
