# Supplementary material for: In Silico Analyses of a Promising Drug Candidate for the Treatment of Amyotrophic Lateral Sclerosis Targeting Superoxide Dismutase I Protein
Source: Pharmaceutics. 2023 Mar 29;15(4):1095. doi: 10.3390/pharmaceutics15041095 (PMC10143751; doi:10.3390/pharmaceutics15041095)
Supplement: Supplementary file 1 [file pharmaceutics-15-01095-s001.zip › Figure_S3.pdf]

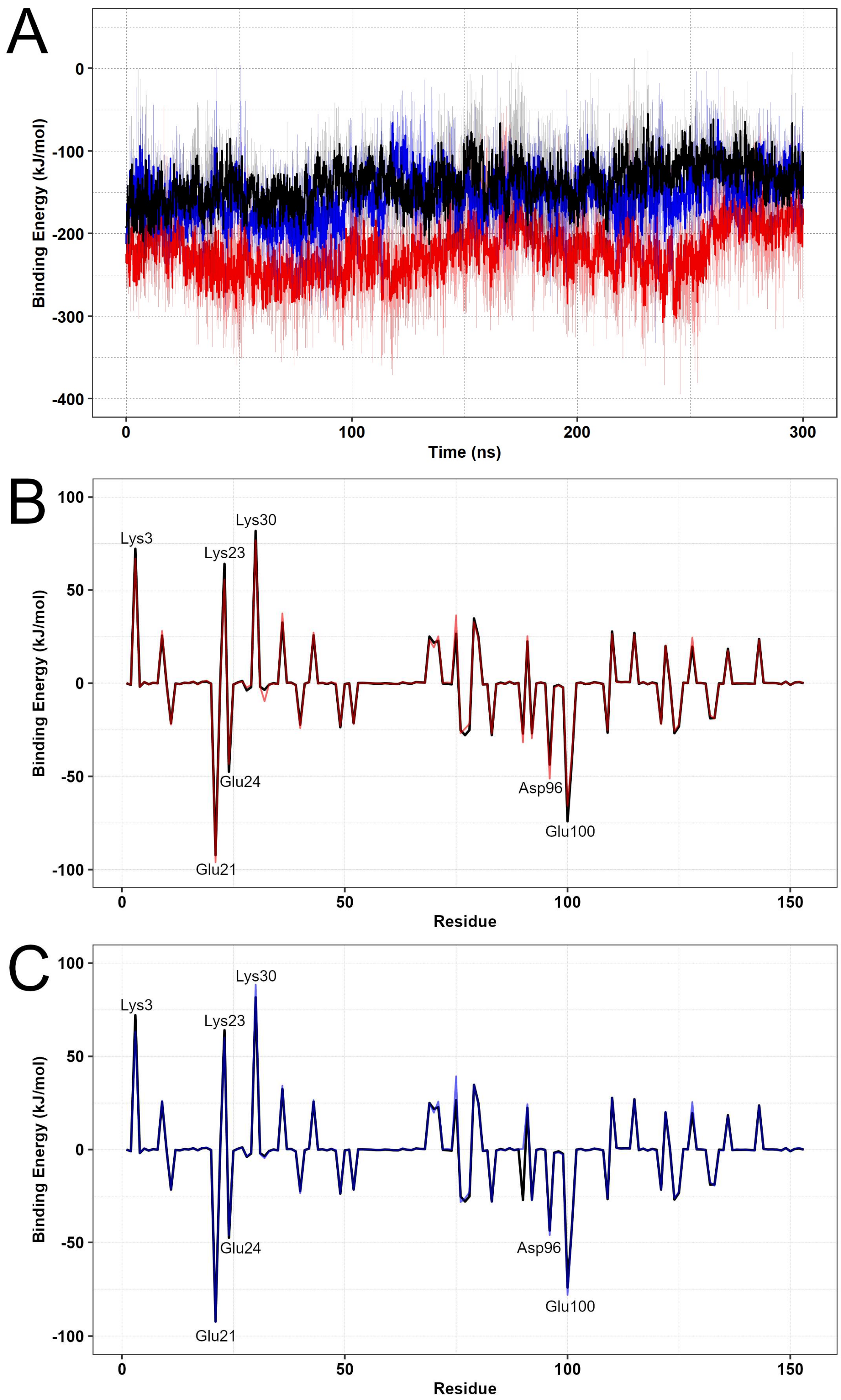

Figure S3. MMPBSA analysis of SOD1-SBL-1 complexes. (**A**) Binding energy values calculated from the interactions of SOD1 wild-type (black) and its variants A4V (red) and D90A (blue) with the SBL-1 compound are shown as a function of time. The means (solid lines) and confidence intervals (smooth lines) are displayed for the triplicates. (**B**) Energetic contribution of each residue of SOD1 wild-type (black) and variant A4V (red) to SBL-1 interaction. (**C**) Energetic contribution of each residue of SOD1 wild-type (black) and variant D90A (blue) to SBL-1 interaction.
