# Supplementary material for: In Silico Analyses of a Promising Drug Candidate for the Treatment of Amyotrophic Lateral Sclerosis Targeting Superoxide Dismutase I Protein
Source: Pharmaceutics. 2023 Mar 29;15(4):1095. doi: 10.3390/pharmaceutics15041095 (PMC10143751; doi:10.3390/pharmaceutics15041095)
Supplement: Supplementary file 1 [file pharmaceutics-15-01095-s001.zip › Table_S1.pdf]

Table S1. Energetic contribution of each SOD1 residue to SBL-1 interaction computed from the MMPBSA analysis.

Residue	Wild-type		A4V		D90A	
	μ	σ	μ	σ	μ	σ
ALA-1	0,00	0,00	0,00	0,00	0,00	0,00
THR-2	-0,91	1,11	-0,72	0,79	-0,97	0,73
LYS-3	72,12	9,14	66,85	5,43	63,34	4,18
ALA-4	-1,88	0,19	-1,97	0,24	-1,87	0,20
VAL-5	0,54	0,14	0,82	0,24	0,56	0,13
CYS-6	-0,55	0,19	-0,74	0,22	-0,59	0,18
VAL-7	0,22	0,13	0,39	0,19	0,24	0,10
LEU-8	-0,07	0,10	-0,17	0,21	-0,06	0,10
LYS-9	25,52	1,67	28,06	2,25	26,23	1,58
GLY-10	-0,13	0,11	-0,24	0,21	-0,17	0,13
ASP-11	-21,46	0,75	-22,19	1,21	-21,33	0,70
GLY-12	-0,17	0,11	-0,23	0,19	-0,19	0,12
PRO-13	-0,47	0,10	-0,60	0,22	-0,49	0,09
VAL-14	0,26	0,08	0,29	0,11	0,28	0,08
GLN-15	0,03	0,70	-0,02	0,92	-0,05	0,69
GLY-16	0,73	0,13	0,97	0,28	0,77	0,13
ILE-17	-0,40	0,25	-0,71	0,40	-0,55	0,25
ILE-18	0,76	0,19	0,76	0,26	0,85	0,22
ASN-19	0,88	1,73	1,43	1,18	1,16	1,03
PHE-20	-0,24	0,47	-0,31	0,61	-0,07	0,49
GLU-21	-92,25	12,72	-96,03	13,96	-92,47	15,27
GLN-22	-3,14	2,32	-3,26	2,98	-2,85	2,02
LYS-23	64,03	10,77	55,55	9,73	60,04	7,61
GLU-24	-47,52	3,17	-43,33	5,78	-46,29	2,95
SER-25	-0,73	0,49	-0,72	0,63	-0,87	0,43
ASN-26	0,42	0,41	0,32	0,52	0,36	0,38
GLY-27	1,19	0,36	0,89	0,41	1,11	0,42
PRO-28	-3,89	2,78	-2,34	2,37	-3,74	4,41
VAL-29	-2,13	1,19	-0,89	1,53	-1,58	1,54
LYS-30	81,67	9,78	76,62	12,40	88,48	11,38
VAL-31	-1,92	0,80	-1,84	0,84	-2,41	0,89
TRP-32	-3,57	4,05	-9,68	3,52	-4,64	3,60
GLY-33	-0,82	0,37	-0,72	0,57	-0,86	0,39
SER-34	0,04	0,77	-0,22	1,05	-0,12	0,72
ILE-35	-0,44	0,14	-0,60	0,23	-0,48	0,15
LYS-36	32,50	3,18	37,42	4,95	34,30	2,25
GLY-37	0,27	0,09	0,34	0,18	0,29	0,10
LEU-38	0,31	0,13	0,16	0,22	0,21	0,18
THR-39	-0,97	0,19	-0,96	0,35	-0,88	0,28
GLU-40	-22,29	0,87	-24,12	1,55	-23,45	0,99
GLY-41	-0,34	0,10	-0,36	0,13	-0,30	0,11
LEU-42	0,56	0,09	0,52	0,15	0,58	0,10
HIS-43	25,69	0,83	27,16	1,16	26,49	0,76

GLY-44	-0,05	0,13	-0,28	0,14	-0,16	0,09
PHE-45	0,16	0,17	0,33	0,19	0,24	0,17
HIS-46	-0,44	0,42	-0,24	0,39	-0,39	0,37
VAL-47	0,35	0,08	0,41	0,10	0,39	0,10
HIS-48	-0,81	0,17	-0,01	0,25	-0,23	0,35
GLU-49	-23,65	0,79	-22,98	1,27	-23,54	0,77
PHE-50	-0,44	0,10	-0,50	0,09	-0,49	0,09
GLY-51	-0,05	0,09	-0,07	0,15	-0,05	0,09
ASP-52	-21,47	0,59	-21,86	0,78	-21,63	0,57
ASN-53	0,11	0,23	-0,04	0,40	0,01	0,37
THR-54	0,14	0,24	0,20	0,35	0,13	0,23
ALA-55	0,05	0,17	0,06	0,13	0,05	0,10
GLY-56	-0,09	0,17	0,05	0,14	0,04	0,18
CYS-57	-0,25	0,14	-0,20	0,16	-0,17	0,17
THR-58	-0,24	0,20	-0,17	0,33	-0,14	0,20
SER-59	-0,01	0,16	-0,15	0,26	0,06	0,26
ALA-60	-0,13	0,13	-0,16	0,14	0,05	0,26
GLY-61	-0,40	0,10	-0,38	0,14	-0,28	0,17
PRO-62	-0,43	0,06	-0,46	0,08	-0,39	0,13
HIS-63	0,41	0,45	0,70	0,40	0,76	0,31
PHE-64	-0,11	0,12	-0,03	0,08	-0,10	0,14
ASN-65	-0,27	0,20	-0,18	0,24	-0,20	0,15
PRO-66	0,61	0,12	0,52	0,18	0,57	0,10
LEU-67	0,40	0,14	0,33	0,16	0,30	0,21
SER-68	0,30	0,24	0,17	0,35	0,15	0,22
ARG-69	25,00	2,24	23,35	1,98	24,90	0,89
LYS-70	21,76	1,39	19,17	1,03	19,78	0,69
HIS-71	22,72	0,67	25,15	1,03	25,78	0,64
GLY-72	-0,15	0,24	0,09	0,11	0,17	0,08
GLY-73	-0,35	0,27	0,43	0,11	0,41	0,10
PRO-74	-0,58	0,26	0,11	0,26	-0,03	0,23
LYS-75	26,54	4,74	36,40	5,03	39,32	4,59
ASP-76	-25,20	1,58	-26,87	1,83	-28,17	0,99
GLU-77	-27,88	3,34	-24,35	2,17	-26,14	1,46
GLU-78	-25,09	1,39	-21,77	1,53	-22,85	0,75
ARG-79	34,75	1,14	32,76	2,34	34,76	1,03
HIS-80	24,93	1,70	23,79	1,10	24,40	0,54
VAL-81	-0,72	0,10	-0,74	0,11	-0,81	0,08
GLY-82	-0,45	0,14	-0,46	0,12	-0,40	0,12
ASP-83	-27,86	1,53	-27,07	1,37	-27,93	0,70
LEU-84	-0,51	0,19	-0,54	0,16	-0,56	0,14
GLY-85	0,32	0,37	-0,11	0,26	-0,03	0,20
ASN-86	-0,35	0,33	-0,37	0,26	-0,38	0,21
VAL-87	0,75	0,12	1,01	0,15	0,96	0,11
THR-88	-0,28	0,38	-0,47	0,43	-0,35	0,37
ALA-89	0,11	0,17	0,29	0,17	0,16	0,12
ASP-90	-26,99	2,68	-31,79	3,69	0,34	0,22

LYS-91	22,37	1,95	25,25	2,68	24,33	1,85
ASP-92	-26,85	1,74	-29,56	2,91	-27,25	1,44
GLY-93	-0,18	0,10	-0,21	0,13	-0,13	0,18
VAL-94	0,38	0,18	0,59	0,27	0,43	0,19
ALA-95	-0,20	0,18	-0,43	0,26	-0,27	0,19
ASP-96	-43,65	3,82	-51,20	7,46	-46,11	4,63
VAL-97	-1,70	0,44	-2,50	0,76	-2,00	0,45
SER-98	-0,92	1,49	-1,09	1,98	-1,58	2,01
ILE-99	-2,29	0,74	-2,26	0,74	-2,63	0,77
GLU-100	-74,10	5,27	-65,83	18,22	-78,04	11,62
ASP-101	-40,64	1,23	-37,93	3,22	-41,04	1,49
SER-102	-0,06	0,78	-0,07	0,68	-0,14	0,84
VAL-103	0,36	0,22	0,28	0,19	0,31	0,15
ILE-104	-0,47	0,19	-0,28	0,26	-0,51	0,20
SER-105	-0,20	0,25	-0,30	0,33	-0,21	0,25
LEU-106	0,53	0,19	0,39	0,18	0,59	0,21
SER-107	0,13	0,45	0,03	0,45	0,14	0,42
GLY-108	-0,41	0,14	-0,41	0,21	-0,38	0,16
ASP-109	-26,58	0,91	-25,30	1,69	-26,16	0,82
HIS-110	27,74	1,89	26,48	2,36	27,84	1,79
CYS-111	0,91	0,35	0,64	0,32	0,81	0,38
ILE-112	0,56	0,13	0,48	0,15	0,56	0,14
ILE-113	0,68	0,14	0,67	0,18	0,67	0,12
GLY-114	0,57	0,09	0,58	0,15	0,57	0,08
ARG-115	27,00	0,74	26,06	1,44	26,89	0,72
THR-116	-0,33	0,28	-0,04	0,16	-0,10	0,17
LEU-117	0,52	0,12	0,58	0,11	0,64	0,11
VAL-118	-0,57	0,12	-0,54	0,12	-0,59	0,11
VAL-119	0,38	0,07	0,39	0,08	0,39	0,07
HIS-120	-0,13	0,34	-0,77	0,25	-0,83	0,16
GLU-121	-21,47	0,72	-21,99	1,05	-21,52	0,64
LYS-122	19,94	0,64	20,20	0,90	20,23	0,74
ALA-123	0,27	0,31	0,49	0,15	0,51	0,10
ASP-124	-26,82	1,24	-25,64	0,99	-26,11	0,83
ASP-125	-23,25	2,22	-22,23	1,02	-22,58	0,75
LEU-126	0,29	0,26	0,18	0,10	0,18	0,08
GLY-127	0,09	0,24	0,13	0,13	0,12	0,08
LYS-128	19,59	2,33	24,48	1,79	25,52	1,19
GLY-129	0,10	0,28	0,20	0,13	0,22	0,07
GLY-130	0,06	0,39	0,24	0,19	0,27	0,09
ASN-131	-0,19	0,43	-0,45	0,21	-0,42	0,20
GLU-132	-18,85	1,31	-17,12	1,00	-17,47	0,55
GLU-133	-18,67	1,05	-18,98	1,03	-19,38	0,75
SER-134	-0,07	0,42	-0,41	0,15	-0,47	0,14
THR-135	-0,24	0,26	-0,42	0,28	-0,45	0,20
LYS-136	18,44	1,10	17,44	0,89	17,79	0,56
THR-137	-0,27	0,32	-0,43	0,29	-0,42	0,20

GLY-138	-0,13	0,16	0,12	0,24	0,21	0,21
ASN-139	-0,15	0,34	-0,21	0,29	-0,16	0,22
ALA-140	-0,10	0,15	-0,08	0,15	0,04	0,14
GLY-141	-0,21	0,34	-0,46	0,18	-0,47	0,09
SER-142	-0,39	0,23	-0,44	0,34	-0,46	0,20
ARG-143	23,68	0,80	23,23	0,80	23,40	0,56
LEU-144	0,31	0,09	0,36	0,12	0,38	0,09
ALA-145	0,07	0,10	0,07	0,11	0,00	0,09
CYS-146	0,10	0,15	0,00	0,16	0,09	0,15
GLY-147	-0,02	0,16	0,01	0,16	-0,06	0,15
VAL-148	-0,50	0,13	-0,70	0,23	-0,55	0,13
ILE-149	0,83	0,11	0,87	0,11	0,86	0,11
GLY-150	-0,91	0,12	-0,89	0,14	-0,95	0,11
ILE-151	0,36	0,19	0,25	0,30	0,44	0,18
ALA-152	0,56	0,35	0,88	0,50	1,06	0,43
GLN-153	0,00	0,00	0,00	0,00	0,00	0,00
