# Supplementary material for: In Silico Analyses of a Promising Drug Candidate for the Treatment of Amyotrophic Lateral Sclerosis Targeting Superoxide Dismutase I Protein
Source: Pharmaceutics. 2023 Mar 29;15(4):1095. doi: 10.3390/pharmaceutics15041095 (PMC10143751; doi:10.3390/pharmaceutics15041095)
Supplement: Supplementary file 1 [file pharmaceutics-15-01095-s001.zip › Table_S2.pdf]

Table S2. ADMET Predictor^{TM} analysis of SBL-1 compound and predicted metabolites.

Structure	ID	Metabolization	ADMET Risk	ABS Risk	CYP Risk	TOX Risk	MUT Risk	MUTx Risk	hERG Toxicity	Acute Toxicity	Carcinog. TOX	REPRO Tox	HEPX	Lipinski's Violations	CYP inhibition
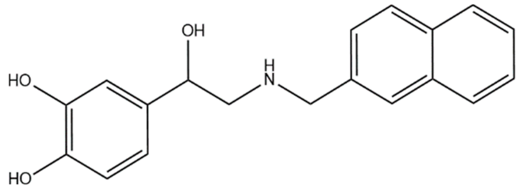	SBL1	–	2,5 [HBD; MUT; 2D6]	0,5 [HBD]	1 [2D6]	1 [MUT]	1,2 [S_97; NIHS]	0,64 [S_97; NIHS]	No	No	No	No	No [AST]	0	CYP1A2, CYP2D6, CYP3A4
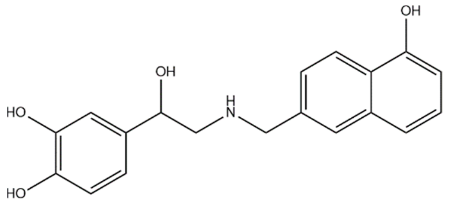	SBL1-M1	Phase I: CYP2D6 [40%]	–	–	–	–	1,2 [S_97; NIHS]	0,64 [S_97; NIHS]	No	–	–	No	–	–	CYP2D6, CYP3A4
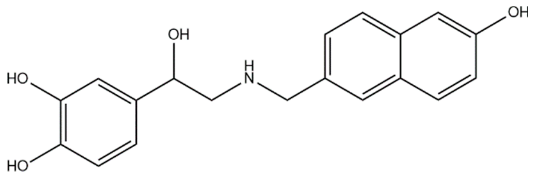	SBL1-M2	Phase I: CYP2D6 [46%]	–	–	–	–	1,2 [S_97; NIHS]	0,64 [S_97; NIHS]	No	–	–	No	–	–	CYP2D6, CYP3A4
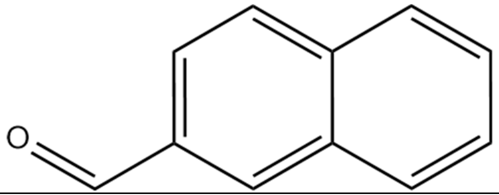	SBL1-M3.1	Phase I: CYP2D6 [14%]	–	–	–	–	1,2 [m_97; NIHS]	0,61 [m_97; NIHS]	No	–	–	No	–	–	CYP1A2, CYP2C19
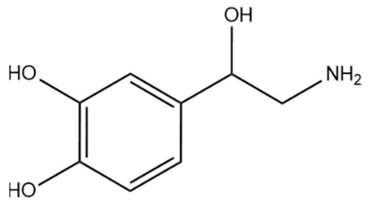	SBL1-M3.2	Phase I: CYP2D6 [14%]	–	–	–	–	0,6 [NIHS]	0,3 [NIHS]	No [hERG-]	–	–	No	–	–	No
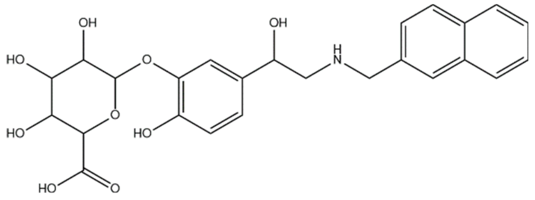	SBL1-M4	Phase II: UGT1A1, UGT1A6, UGT1A9, UGT1A10, UGT2B7	–	–	–	–	0,6 [S_97]	0,34 [S_97]	No [hERG-]	–	–	No	–	–	CYP3A4

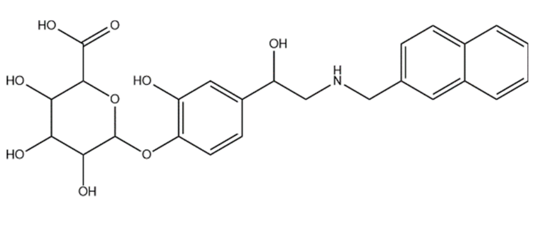	SBL1-M5	Phase II: UGT1A1, UGT1A6, UGT1A9, UGT1A10, UGT2B7	–	–	–	–	0,6 [S_97]	0,34 [S_97]	No [hERG-]	–	–	No	–	–	CYP3A4
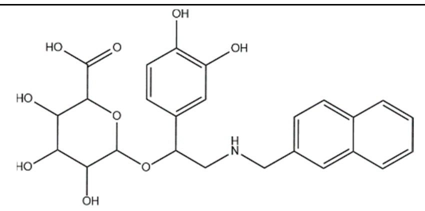	SBL1-M6	Phase II: UGT1A3	–	–	–	–	1,2 [S_97; NIHS]	0,64 [S_97; NIHS]	No [hERG-]	–	–	No	–	–	CYP3A4
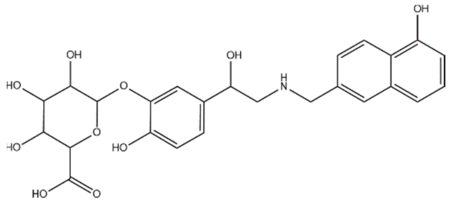	SBL1-M1-M1	Phase I: CYP2D6; Phase II: UGT1A1, UGT1A6, UGT1A9, UGT1A10, UGT2B7	–	–	–	–	0,6 [S_97]	0,34 [S_97]	No [hERG-]	–	–	No	–	–	CYP3A4
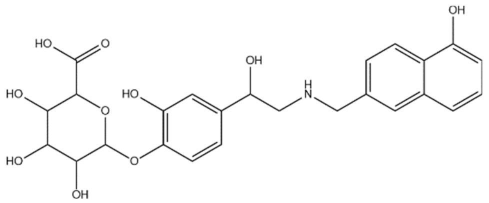	SBL1-M1-M2	Phase I: CYP2D6; Phase II: UGT1A1, UGT1A6, UGT1A9, UGT1A10, UGT2B7	–	–	–	–	0,6 [S_97]	0,34 [S_97]	No [hERG-]	–	–	No	–	–	CYP3A4
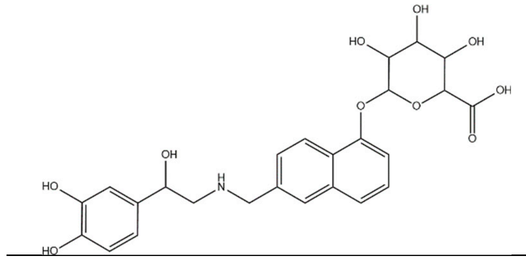	SBL1-M1-M3	Phase I: CYP2D6; Phase II: UGT1A1, UGT1A6, UGT1A9, UGT1A10, UGT2B7	–	–	–	–	0,6 [S_97]	0,34 [S_97]	No [hERG-]	–	–	No	–	–	CYP3A4
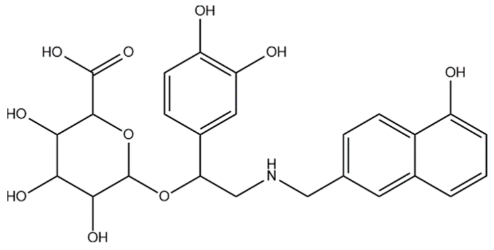	SBL1-M1-M4	Phase I: CYP2D6; Phase II: UGT1A3	–	–	–	–	1,2 [S_97; NIHS]	0,64 [S_97; NIHS]	No [hERG-]	–	–	No	–	–	CYP3A4

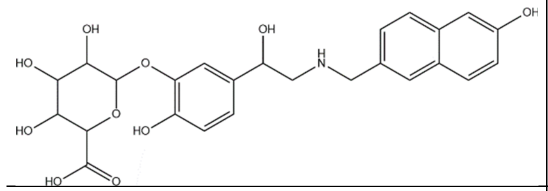	SBL1-M2-M1	Phase I: CYP2D6; Phase II: UGT1A1, UGT1A6, UGT1A9, UGT1A10, UGT2B7	–	–	–	–	0,6 [S_97]	0,34 [S_97]	No [hERG-]	–	–	No	–	–	CYP3A4
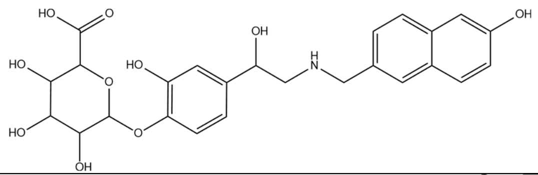	SBL1-M2-M2	Phase I: CYP2D6; Phase II: UGT1A1, UGT1A6, UGT1A9, UGT1A10, UGT2B7	–	–	–	–	0,6 [S_97]	0,34 [S_97]	No [hERG-]	–	–	No	–	–	CYP3A4
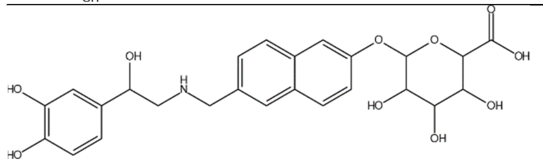	SBL1-M2-M3	Phase I: CYP2D6; Phase II: UGT1A1, UGT1A6, UGT1A9, UGT1A10, UGT2B7	–	–	–	–	0,6 [S_97]	0,34 [S_97]	No [hERG-]	–	–	No	–	–	CYP3A4
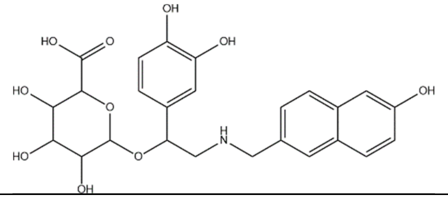	SBL1-M2-M4	Phase I: CYP2D6; Phase II: UGT1A3	–	–	–	–	1,2 [S_97; NIHS]	0,64 [S_97; NIHS]	No [hERG-]	–	–	No	–	–	CYP3A4
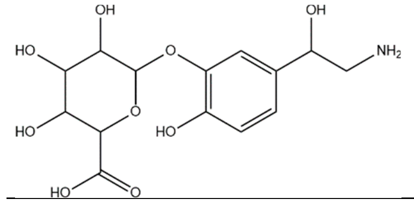	SBL1-M3.2-M1	Phase I: CYP2D6; Phase II: UGT1A6	–	–	–	–	0	0	No [hERG-]	–	–	No	–	–	CYP3A4
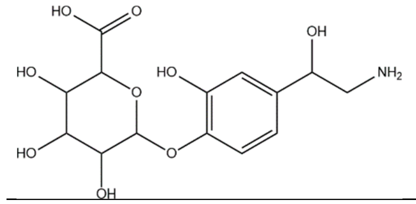	SBL1-M3.2-M2	Phase I: CYP2D6; Phase II: UGT1A6	–	–	–	–	0	0	No [hERG-]	–	–	No	–	–	CYP3A4

The respective ADMET-rule violation is shown between brackets. HBD: hydrogen bond donor; HEPX: hepatotoxicity; MUT: mutagenic; 2D6: increased intrinsic clearance for CYP2D6; S_97: mutagenic in *S. Typhimurium* TA97+TA1537 strains without microsomal activation; NIHS: classified as mutagenic by a model trained on the NIHS's Ames test dataset; hERG-: out-of-scope cardiotoxicity in rats; AST: elevated levels of aspartic acid transaminase in serum; Underline ("_") represents analysis not performed for the metabolites.
