# Supplementary material for: In Silico Analyses of a Promising Drug Candidate for the Treatment of Amyotrophic Lateral Sclerosis Targeting Superoxide Dismutase I Protein
Source: Pharmaceutics. 2023 Mar 29;15(4):1095. doi: 10.3390/pharmaceutics15041095 (PMC10143751; doi:10.3390/pharmaceutics15041095)
Supplement: Supplementary file 1 [file pharmaceutics-15-01095-s001.zip › Table_S3.pdf]

Table S3. Percepta analysis of SBL-1 compound and predicted metabolites.

Structure	ID	Acute toxicity	Mutagenicity	hERG inhibition	CYP450 inhibition*	BBB penetration	PysChem profiling	ADME profiling
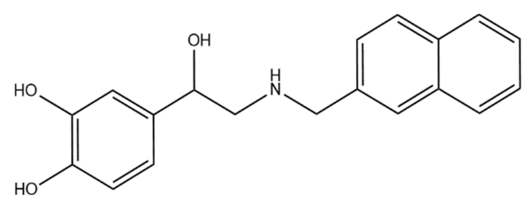	SBL1	No hazardous fragment	0.35 (Undefined) [Epinephrine, Isoproterenol, Phenylephrine, Nylidrin, Pronethalol]	0.31 (Non-inhibitor) [Isoproterenol, Morin, Caffeic acid phenethyl ester, Naringenin]	CYP3A4: 0.4 (Undefined)<br>CYP2D6: 0.64 (Undefined)<br>CYP2C9: 0.33 (Non-inhibitor)<br>CYP2C19: 0.38 (Undefined)<br>CYP1A2: 0.48 (Undefined)	LogBB: 0.17;<br>Log(PS*fu, brain): -3.1;<br>CNS score: -2.93 (Sufficient for CNS activity)	Lipinski's violations: 0;<br>Lead-like violations: 0;<br>Solubility: 33.9mg/ml (Soluble)<br>Rotatable bonds: 5;<br>Nº of rings: 3	Bioavailability: 96%;<br>Caco-2:16E-6cm/s(High);<br>HIA: 100%(High);<br>PPB: 81% (Good);<br>Metabolic Stability: 0.49 (Undefined)
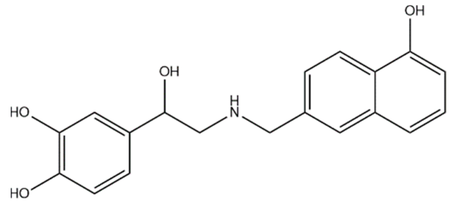	M1	–	0.32 (Non-mutagenic) [Epinephrine, Isoproterenol, Phenylephrine, Albuterol, Nylidrin]	0.31 (Non-inhibitor) [Isoproterenol, Morin, Naringenin, Caffeic acid phenethyl ester]	CYP3A4: 0.38 (Undefined)<br>CYP2D6: 0.52 (Undefined)<br>CYP2C9: 0.33 (Non-inhibitor)<br>CYP2C19: 0.38 (Undefined)<br>CYP1A2: 0.44 (Undefined)	–	–	–
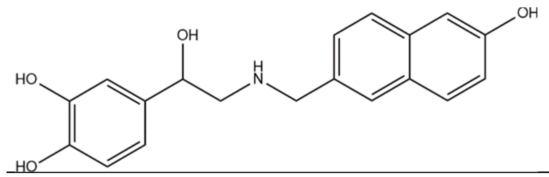	M2	–	0.32 (Non-mutagenic) [Epinephrine, Isoproterenol, Phenylephrine, Albuterol, Nylidrin	0.31 (Non-inhibitor) [Isoproterenol, Morin, Naringenin, Caffeic acid phenethyl ester]	CYP3A4: 0.36 (Undefined)<br>CYP2D6: 0.59 (Undefined)<br>CYP2C9: 0.33 (Non-inhibitor)<br>CYP2C19: 0.36 (Undefined)<br>CYP1A2: 0.43 (Undefined)	–	–	–
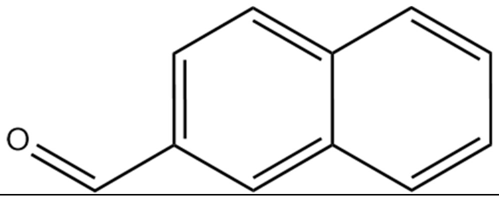	M3.1	–	0.44 (Undefined) [Benzaldehyde, Biphenyl-carboxaldehyde, Anthraldehyde, Tolualdehyde]	0.42 (Undefined) [Fampiridine, Arecoline, Methyl ecgonidine]	CYP3A4: 0.26 (Non-inhibitor)<br>CYP2D6: 0.38 (Undefined)<br>CYP2C9: 0.36 (Undefined)<br>CYP2C19: 0.42 (Undefined)<br>CYP1A2: 0.57 (Undefined)	–	–	–
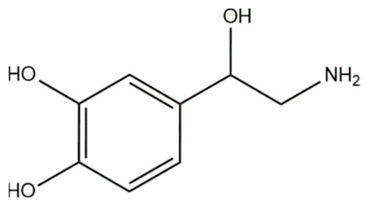	M3.2	–	0.48 (Undefined) [Epinephrine, Isoproterenol]	0.32 (Non-inhibitor) [Isoproterenol, Morin, Naringenin, Hesperetin]	CYP3A4: 0.07 (Non-inhibitor)<br>CYP2D6: 0.09 (Non-inhibitor)<br>CYP2C9: 0.08 (Non-inhibitor)<br>CYP2C19: 0.09 (Non-inhibitor)<br>CYP1A2: 0.09 (Non-inhibitor)	–	–	–
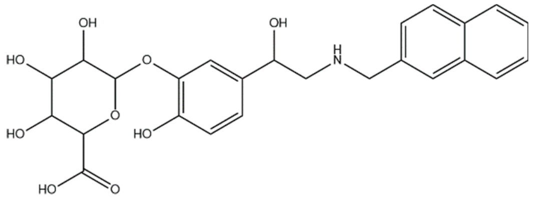	M4	–	0.4 (Undefined) [Naphthylacetic acid, Butenafine, Hydrocinnamic acid]	0.35 (Undefined) [Isoproterenol, Morin, Adriamycin, ouabain]	CYP3A4: 0.24 (Non-inhibitor)<br>CYP2D6: 0.3 (Non-inhibitor)<br>CYP2C9: 0.22 (Non-inhibitor)<br>CYP2C19: 0.19 (Non-inhibitor)<br>CYP1A2: 0.14 (Non-inhibitor)	–	–	–
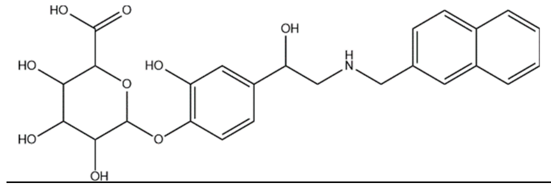	M5	–	0.4 (Undefined) [Naphthylacetic acid, Glycyrrhizic acid, Fosinopril, Hydrocinnamic acid, Ephedrine]	0.34 (Undefined) [Isoproterenol, Morin, Ouabain, Adriamycin]	CYP3A4: 0.23 (Non-inhibitor)<br>CYP2D6: 0.3 (Non-inhibitor)<br>CYP2C9: 0.21 (Non-inhibitor)<br>CYP2C19: 0.2 (Non-inhibitor)<br>CYP1A2: 0.14 (Non-inhibitor)	–	–	–

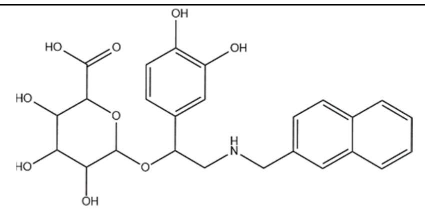	M6	–	0.39 (Undefined) [Okadaic acid, Delapril, Glycyrrhizin acid, Enalaprilat]	0.36 (Undefined) [Isoproterenol, Morin, Naringenin, Ouabain]	CYP3A4: 0.26 (Non-inhibitor) CYP2D6: 0.33 Non-inhibitor CYP2C9: 0.27 (Non-inhibitor) CYP2C19: 0.25 (Non-inhibitor) CYP1A2: 0.18 (Non-inhibitor)	–	–	–
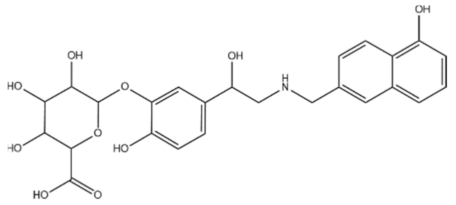	M1-M1	–	0.41 (Undefined) [Naphthyl acetic acid, Dihydroxyphenyl acetic acid, Isoproterenol]	0.34 (Undefined) [Isoproterenol, Morin, Ouabain, Adriamycin]	CYP3A4: 0.27 (Non-inhibitor) CYP2D6: 0.32 (Non-inhibitor) CYP2C9: 0.25 (Non-inhibitor) CYP2C19: 0.2 (Non-inhibitor) CYP1A2: 0.15 (Non-inhibitor)	–	–	–
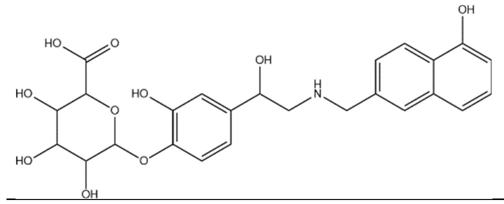	M1-M2	–	0.41 (Undefined) [Isoproterenol, Phenylephrine, Nylidrin, Epinephrine]	0.33 (Non-inhibitor) [Isoproterenol, Morin, Ouabain, Adriamycin]	CYP3A4: 0.27 (Non-inhibitor) CYP2D6: 0.32 (Non-inhibitor) CYP2C9: 0.25 (Non-inhibitor) CYP2C19: 0.21 (Non-inhibitor) CYP1A2: 0.16 (Non-inhibitor)	–	–	–
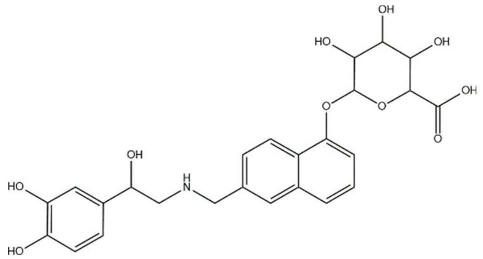	M1-M3	–	0.37 (Undefined) [Phenylephrine, Epinephrine, Isoproterenol, Ephedrine, Albuterol]	0.33 (Non-inhibitor) [Isoproterenol, Morin, Ouabain, Epirubicin]	CYP3A4: 0.27 (Non-inhibitor) CYP2D6: 0.32 (Non-inhibitor) CYP2C9: 0.22 (Non-inhibitor) CYP2C19: 0.21 (Non-inhibitor) CYP1A2: 0.14 (Non-inhibitor)	–	–	–
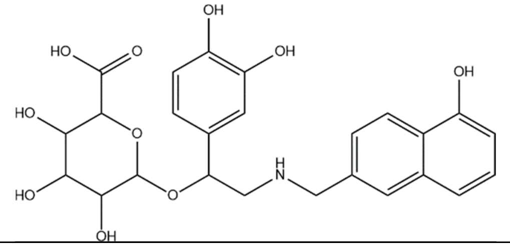	M1-M4	–	0.42 (Undefined) [Isoproterenol, Phenylephrine, Trandolapril]	0.35 (Undefined) [Isoproterenol, Morin, Naringenin, Hesperetin]	CYP3A4: 0.29 (Non-inhibitor) CYP2D6: 0.35 (Undefined) CYP2C9: 0.31 (Non-inhibitor) CYP2C19: 0.26 (Non-inhibitor) CYP1A2: 0.21 (Non-inhibitor)	–	–	–
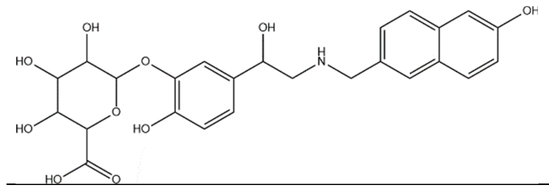	M2-M1	–	0.41 (Undefined) [Isoproterenol, Naphthyl acetic acid, Dihydroxyphenyl acetic acid]	0.34 (Undefined) [Isoproterenol, Morin, Ouabain, Adriamycin]	CYP3A4: 0.24 (Non-inhibitor) CYP2D6: 0.31 (Non-inhibitor) CYP2C9: 0.22 (Non-inhibitor) CYP2C19: 0.19 (Non-inhibitor) CYP1A2: 0.15 (Non-inhibitor)	–	–	–
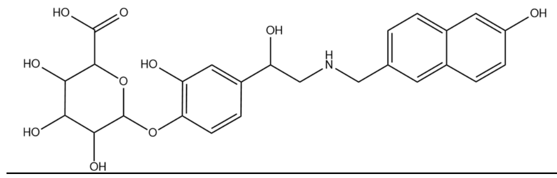	M2-M2	–	0.41 (Undefined) [Epinephrine, isoproterenol, phenylephrine, nylidrin]	0.33 (Non-inhibitor) [Isoproterenol, Morin, Ouabain, Adriamycin]	CYP3A4: 0.24 (Non-inhibitor) CYP2D6: 0.31 (Non-inhibitor) CYP2C9: 0.22 (Non-inhibitor) CYP2C19: 0.2 (Non-inhibitor) CYP1A2: 0.15 (Non-inhibitor)	–	–	–

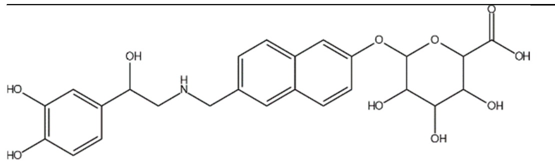	M2-M3	–	0.4 (Undefined) [Epinephrine, Isoproterenol, Phenylephrine, Dihydroxyphenyl acetic acid]	0.33 (Non-inhibitor) [Isoproterenol, Morin, Ouabain, Epirubicin]	CYP3A4: 0.27 (Non-inhibitor)<br>CYP2D6: 0.33 (Non-inhibitor)<br>CYP2C9: 0.26 (Non-inhibitor)<br>CYP2C19: 0.21 (Non-inhibitor)<br>CYP1A2: 0.16 (Non-inhibitor)	–	–	–
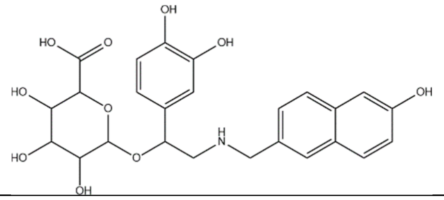	M2-M4	–	0.42 (Undefined) [isoproterenol, phenylephrine, trandalopril]	0.35 (Undefined) [Isoproterenol, Morin, Naringenin, Hesperetin]	CYP3A4: 0.24 (Non-inhibitor)<br>CYP2D6: 0.33 (Non-inhibitor)<br>CYP2C9: 0.29 (Non-inhibitor)<br>CYP2C19: 0.25 (Non-inhibitor)<br>CYP1A2: 0.2 (Non-inhibitor)	–	–	–
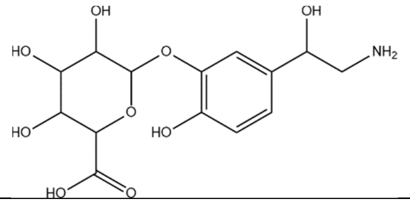	M3.2-M1	–	0.44 (Undefined) [Epinephrine, alpha-Methyldopa, Isoproterenol, Dihydroxyphenyl acetic acid]	0.3 (Non-inhibitor) [Isoproterenol, Ouabain, Morin, Epirubicin]	CYP3A4: 0.19 (Non-inhibitor)<br>CYP2D6: 0.21 (Non-inhibitor)<br>CYP2C9: 0.14 (Non-inhibitor)<br>CYP2C19: 0.18 (Non-inhibitor)<br>CYP1A2: 0.1 (Non-inhibitor)	–	–	–
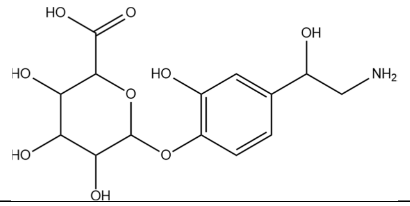	M3.2-M2	–	0.44 (Undefined) [Epinephrine, alpha-Methyldopa, Isoproterenol, Dihydroxyphenyl acetic acid]	0.3 (Non-inhibitor) [Isoproterenol, Ouabain, Morin, Epirubicin]	CYP3A4: 0.19 (Non-inhibitor)<br>CYP2D6: 0.21 (Non-inhibitor)<br>CYP2C9: 0.14 (Non-inhibitor)<br>CYP2C19: 0.18 (Non-inhibitor)<br>CYP1A2: 0.1 (Non-inhibitor)	–	–	–

Numeric values corresponding to the scores estimated by ACD/Percepta predictors are shown followed by their corresponding classification between parentheses when applied. Structurally related compounds with similarly defined classifications are provided between brackets when experimental evidence is available. For undefined classifications, only compounds with favorable classifications are shown when experimental evidence is available, *i.e*, non-inhibitor, non-mutagenic, and non-toxic.

*Detailed information concerning CYP inhibition analysis is provided in S4_Table.
