# Supplementary material for: In Silico Analyses of a Promising Drug Candidate for the Treatment of Amyotrophic Lateral Sclerosis Targeting Superoxide Dismutase I Protein
Source: Pharmaceutics. 2023 Mar 29;15(4):1095. doi: 10.3390/pharmaceutics15041095 (PMC10143751; doi:10.3390/pharmaceutics15041095)
Supplement: Supplementary file 1 [file pharmaceutics-15-01095-s001.zip › Table_S4.pdf]

Table S4. CYP inhibition analysis of SBL-1 compound and predicted metabolites performed using the ACD/Percepta platform.

Structure	ID	CYP3A4 inhibition	CYP2D6 inhibition	CYP2C9 inhibition	CYP2C19 inhibition	CYP1A2 inhibition
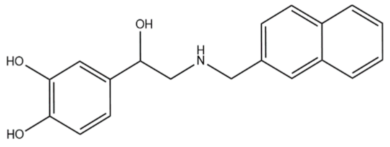	SBL1	0.4 (Undefined) [Epinephrine, Levonordefrin, Norepinephrine]	0.64 (Undefined) [Epinephrine, Isoproterenol, Deoxynephrine]	0.33 (Non-inhibitor) [Epinephrine, Isoproterenol]	0.38 (Undefined) [Denopamine, Epinephrine, Isoproterenol]	0.48 (Undefined) [Denopamine, Benzamide]
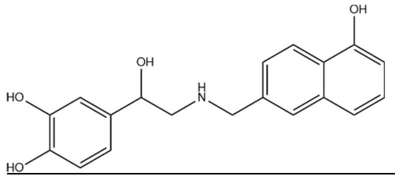	M1	0.38 (Undefined) [Epinephrine, Deoxynephrine]	0.52 (Undefined) [Epinephrine, Isoproterenol, Deoxynephrine]	0.33 (Non-inhibitor) [Epinephrine, Isoproterenol]	0.38 (Undefined) [Epinephrine, Isoproterenol, Norepinephrine]	0.44 (Undefined) [Epinephrine]
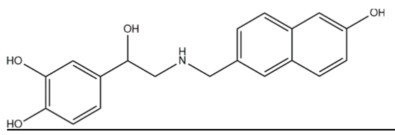	M2	0.36 (Undefined) [Epinephrine, Norepinephrine, Levonordefrin, Ethylnorepinephrine]	0.59 (Undefined) [Epinephrine, Isoproterenol, Deoxynephrine]	0.33 (Non-inhibitor) [Epinephrine, Isoproterenol]	0.36 (Undefined) [Epinephrine, Isoproterenol, Denopamine]	0.43 (Undefined) [Denopamine, Benzamide]
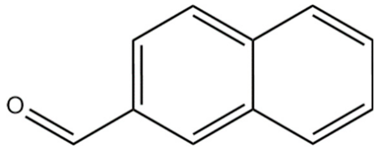	M3.1	0.26 (Non-inhibitor) [Napthtalene, a-Methylnaphtalene, b-Methylnaphtalene, Nitronaphtalene, 4-Methylthiobenzaldehyde	0.38 (Undefined) [Indole 3-acetaldehyde, 4-Nitrobenzoyl-chloride, Quinone]	0.36 (Undefined) [Nabumetone, Benzene]	0.42 (Undefined) [Diacetyl, 1-Methylimidazole]	0.57 (Undefined) [Naphthalene, 2-Ethynylnaphthalene, Quinoline]
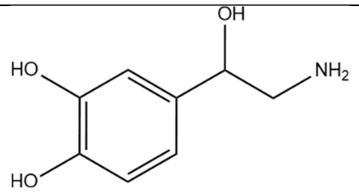	M3.2	0.07 (Non-inhibitor) [Norepinephrine, Levonordefrin, Ethylnorepinephrine, Epinephrine, Dopamine]	0.09 (Non-inhibitor) [Norepinephrine, Levonordefrin, Metaraminol]	0.08 (Non-inhibitor) [Norepinephrine, Levonordefrin, Dopamine]	0.09 (Non-inhibitor) [Norepinephrine, Levonordefrin, Metaraminol]	0.09 (Non-inhibitor) [Norepinephrine, Levonordefrin]
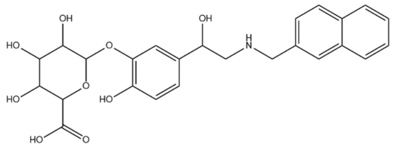	M4	0.24 (Non-inhibitor) [Talibegron, 4-Methylumbelliferyl Glucuronide]	0.3 (Non-inhibitor)	0.22 (Non-inhibitor)	0.19 (Non-inhibitor)	0.14 (Non-inhibitor)
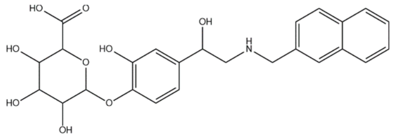	M5	0.23 (Non-inhibitor) [Talibegron, 4-Methylumbelliferyl Glucuronide]	0.3 (Non-inhibitor) [Epinephrine, DL-Dopa]	0.21 (Non-inhibitor)	0.2 (Non-inhibitor)	0.14 (Non-inhibitor)

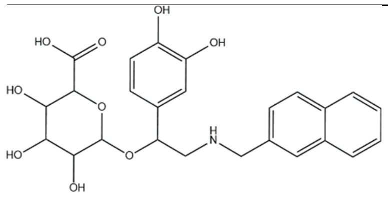	M6	0.26 (Non-inhibitor) [Chlorogenic acid, 1-Naphthylacetic acid, Benzenepropanoic acid]	0.33 Non-inhibitor [DL-Dopa, 3,4-Dihydroxycinnamic Acid]	0.27 (Non-inhibitor) [Isoprotereno, Epinephrinel]	0.25 (Non-inhibitor) [Ginsenoside]	0.18 (Non-inhibitor)
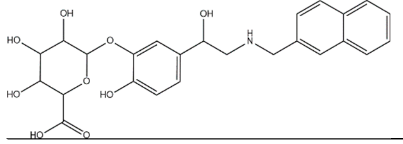	M1-M1	0.27 (Non-inhibitor) [Talibegron, Chlorogenic acid, 1-Naphthylacetic acid]	0.32 (Non-inhibitor) [Epinephrine, (3,4-Dihydroxyphenyl)acetic acid, DL-Dopa]	0.25 (Non-inhibitor)	0.2 (Non-inhibitor)	0.15 (Non-inhibitor)
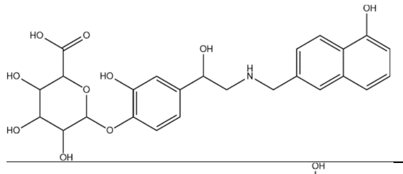	M1-M2	0.27 (Non-inhibitor) [Talibegron, Chlorogenic acid, 1-Naphthylacetic acid]	0.32 (Non-inhibitor) [Epinephrine, (3,4-Dihydroxyphenyl) acetic acid, DL-Dopa]	0.25 (Non-inhibitor)	0.21 (Non-inhibitor)	0.16 (Non-inhibitor)
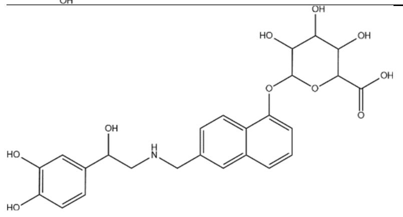	M1-M3	0.27 (Non-inhibitor) [Talibegron, Chlorogenic acid, 1-Naphthylacetic acid]	0.32 (Non-inhibitor) [Epinephrine, (3,4-Dihydroxyphenyl)acetic acid, DL-Dopa, Isoproterenol]	0.22 (Non-inhibitor)	0.21 (Non-inhibitor)	0.14 (Non-inhibitor)
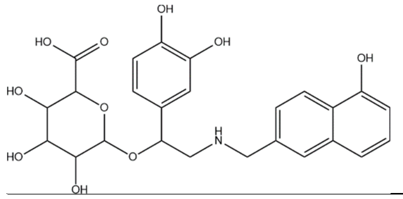	M1-M4	0.29 (Non-inhibitor) [(3,4-Dihydroxyphenyl) acetic acid, DL-Dopa, Chlorogenic acid]	0.35 (Undefined) [DL-Dopa, (3,4-Dihydroxyphenyl)acetic acid, 3,4-Dihydroxycinnamic acid, Deoxyepinephrine]	0.31 (Non-inhibitor) [Epinephrine, Isoproterenol]	0.26 (Non-inhibitor) [Ginsenoside]	0.21 (Non-inhibitor)
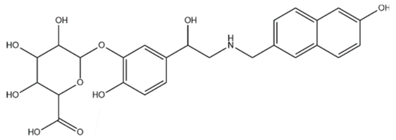	M2-M1	0.24 (Non-inhibitor) [Talibegron, 1-Naphthylacetic acid]	0.31 (Non-inhibitor) [Epinephrine, DL-Dopa, (3,4-Dihydroxyphenyl)acetic acid]	0.22 (Non-inhibitor)	0.19 (Non-inhibitor)	0.15 (Non-inhibitor)
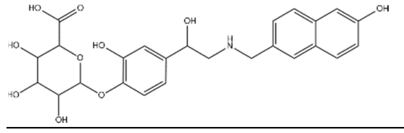	M2-M2	0.24 (Non-inhibitor) [Talibegron, Chlorogenic acid]	0.31 (Non-inhibitor) [Epinephrine, DL-Dopa, (3,4-Dihydroxyphenyl)acetic acid]	0.22 (Non-inhibitor)	0.2 (Non-inhibitor)	0.15 (Non-inhibitor)

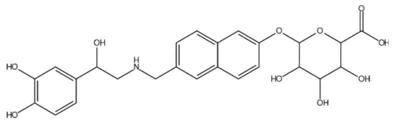	M2-M3	0.27 (Non-inhibitor) [Talibegron, Chlorogenic acid, 1-Naphthylacetic acid]	0.33 (Non-inhibitor) [Epinephrine, (3,4-Dihydroxyphenyl)acetic acid, DL-Dopa, Isoproterenol]	0.26 (Non-inhibitor)	0.21 (Non-inhibitor)	0.16 (Non-inhibitor)
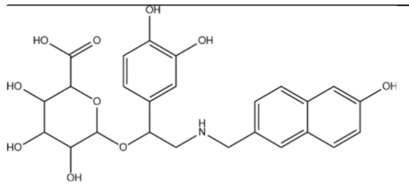	M2-M4	0.24 (Non-inhibitor) [Chlorogenic acid, DL-Dopa, (3,4-Dihydroxyphenyl)acetic acid]	0.33 (Non-inhibitor) [DL-Dopa, 3,4-Dihydroxycinnamic Acid, (3,4-Dihydroxyphenyl) acetic acid]	0.29 (Non-inhibitor) [Epinephrine, Isoproterenol]	0.25 (Non-inhibitor)	0.2 (Non-inhibitor)
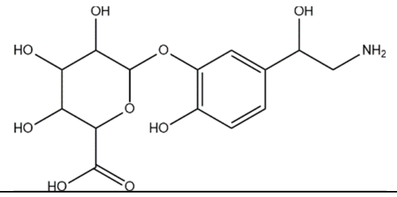	M3.2-M1	0.19 (Non-inhibitor) [D-Glucuronic acid, D-2,3,4,5-Tetrahydroxyhexanedioic acid]	0.21 (Non-inhibitor) [DL-Dopa, Vanilmandelic acid]	0.14 (Non-inhibitor) [Norepinephrine]	0.18 (Non-inhibitor)	0.1 (Non-inhibitor)
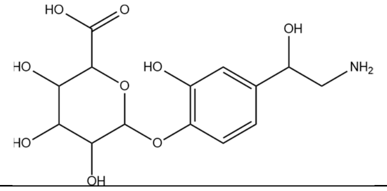	M3.2-M2	0.19 (Non-inhibitor) [D-Glucuronic acid, D-2,3,4,5-Tetrahydroxyhexanedioic acid]	0.21 (Non-inhibitor) [DL-Dopa, Vanilmandelic acid]	0.14 (Non-inhibitor) [Norepinephrine]	0.18 (Non-inhibitor)	0.1 (Non-inhibitor)

Numeric values corresponding to the scores estimated by CYP inhibition predictors are shown followed by their corresponding classification between parentheses. Structurally related compounds with similarly defined classifications are provided between brackets when experimental evidence is available. For undefined classifications, only compounds with non-inhibitor classification are shown when experimental evidence is available.
